# Understanding rare genetic variants within the terminal pathway of complement system in preeclampsia

**DOI:** 10.1038/s41435-024-00310-6

**Published:** 2024-12-17

**Authors:** A. Inkeri Lokki, Michael Triebwasser, Emma Daly, The FINNPEC Core Investigator Group, Seppo Heinonen, Eero Kajantie, Juha Kere, Katja Kivinen, Anneli Pouta, Hannele Laivuori, Mitja I. Kurki, Markus Perola, Kirsi Auro, Jane E. Salmon, Java Anuja, Mark Daly, John P. Atkinson, Hannele Laivuori, Seppo Meri

**Affiliations:** 1 Translational Immunology Research Program, Research Programs Unit and Bacteriology and Immunology, University of Helsinki and Helsinki University Hospital, Helsinki, Finland; 2 Heart and Lung Center, Helsinki University Central Hospital and University of Helsinki, Helsinki, Finland; 3 Department of Pediatrics, Division of Pediatric Hematology and Oncology, University of Michigan, Ann Arbor, MI USA; 4 Hospital and Harvard Medical School, Boston, MA USA; 5 Institute for Molecular Medicine Finland, Helsinki Institute of Life Science, University of Helsinki, Helsinki, Finland; 6 Analytic and Translational Genetics Unit, Department of Medicine, Massachusetts General Hospital and Harvard Medical School, Boston, MA USA; 7 Stanley Center for Psychiatric Research, Broad Institute of MIT and Harvard, Cambridge, MA USA; 8 Department of Public Health and Welfare, National Institute for Health and Welfare, Helsinki, Finland; 9 Hospital for Special Surgery; Weill Medical College of Cornell University, New York, NY USA; 10 Division of Nephrology, Department of Medicine, Washington University School of Medicine in St. Louis, St. Louis, MO USA; 11 Analytic and Translational Genetics Unit, Massachusetts General Hospital, Boston, MA USA; 12 Division of Rheumatology, Department of Medicine, Washington University School of Medicine, St. Louis, MO USA; 13 Medical and Clinical Genetics, University of Helsinki and Helsinki University Hospital, Helsinki, Finland; 14 Department of Obstetrics and Gynecology, Tampere University Hospital and the Wellbeing Services County of Pirkanmaa, Tampere, Finland; 15 Center for Child, Adolescent, and Maternal Health Research, Faculty of Medicine and Health Technology, Tampere University, Tampere, Finland; 16 HUSLAB Diagnostic Center, Helsinki University Central Hospital, Helsinki, Finland; 17 Obstetrics and Gynecology, University of Helsinki and Helsinki University Hospital, Helsinki, Finland; 18 Research Unit of Clinical Medicine and Medical Research Centre Oulu, University of Oulu, Oulu, Finland; 19 Department of Paediatrics and Adolescent Medicine, Oulu University Hospital, Oulu, Finland; 20 Population Health Unit, Finnish Institute for Health and Welfare, Helsinki, Finland; 21 Department of Clinical and Molecular Medicine, Norwegian University of Science and Technology, Trondheim, Norway; 22 Department of Biosciences and Nutrition, Karolinska Institutet, Huddinge, Sweden; 23 Folkhälsan Research Center, Helsinki, Finland; 24 School of Basic & Medical Biosciences, King’s College London, London, United Kingdom; 25 Division of Cardiovascular Medicine, University of Cambridge, Cambridge, United Kingdom; 26 Department of Government Services, National Institute for Health and Welfare, Helsinki, Finland

**Keywords:** Genetic association study, Complement cascade

## Abstract

Preeclampsia is a common multifactorial disease of pregnancy. Dysregulation of complement activation is among emerging candidates responsible for disease pathogenesis. In a targeted exomic sequencing study of 609 women with preeclampsia and 2092 non-preeclamptic controls, we identified 14 variants within nine genes coding for components of the membrane attack complex (MAC, C5b-9) that are associated with preeclampsia. We found two rare missense variants in the *C5* gene that predispose to preeclampsia (rs200674959: I1296V, OR (CI95) = 24.13 (1.25–467.43), *p* value = 0.01 and rs147430470: I330T, OR (CI95) = 22.75 (1.17–440.78), *p* value = 0.01). In addition, one predisposing rare variant and one protective rare variant were discovered in *C6* (rs41271067: D396G, OR (CI95) = 2.93 (1.18–7.10), *p* value = 0.01 and rs114609505: T190I, 0.02 OR (CI95) = 0.47 (0.22–0.92), *p* value = 0.02). The results suggest that variants in the terminal complement pathway predispose to preeclampsia.

## Introduction

Preeclampsia (PE) is a common pregnancy-specific vascular disorder that affects approximately 3% of pregnancies [1, 2]. It accounts for over 50 000 maternal and 900 000 perinatal deaths annually [3, 4]. The clinical characteristics are diverse and the course of the disease is unpredictable. The cornerstones of management are observation and delivery. Even when high quality antenatal healthcare is available, maternal morbidity is considerable and induced preterm deliveries may result in newborn complications. There is a familial predisposition to PE and strong epidemiological evidence points to PE being partially inherited [5, 6]. Furthermore, loci harbouring genes that have inflammatory or immune regulatory function were recently found to link PE and hypertension in pregnancy [7, 8].

The complement system is at the frontline of innate immunity with a capacity to cause cell death and tissue destruction as well as to trigger adaptive immune responses. In particular, the complement system has a unique capacity to discriminate between self and non-self structures and to recognize nonviable cells [9]. Inadequate regulation may result in poor placentation and predispose to the development of PE [10–12]. Activation of any of the three pathways of the complement system, the classical (CP), lectin (LP) or alternative pathway (AP), can lead to a common end point: terminal pathway activation featuring a membrane attack complex (MAC, C5b-9) formation. Insertion of the MAC in the plasma membrane is initiated by the C5b-8 complex composed of the C5b, C6, C7 and C8 [13]. After cleavage of C5, the generated soluble C5b binds C6, which then recruits C7 and C5b67 can attach to a membrane. Further binding of C8 leads to insertion of the C5b-8 complex into a membrane. This step enables recruitment of multiple C9 molecules to complete the cylindrical MAC structure [14]. In the MAC pore, C9 proteins constitute a polymeric ring with an inner diameter of about 10 nm [15]. Because of a risk of membrane damage during complement activation, human cells are protected from damage by protectin (CD59). This GPI anchored protein inhibits MAC formation by binding to C5b-8 and C5b-9 to prevent further C9 from attaching to these complexes [16, 17]. Therefore, the balanced regulatory capacity of CD59 and MAC activation is crucial for integrity and function of endothelial surfaces and placental trophoblast cells (CD59 is highly expressed in both of these cell types) [18].

The major activators of the CP are antibody-antigen complexes and C-reactive protein (CRP). Subsequent proteolytic activation steps by C1 lead to cleavage of C4 and C2 and formation of the CP C3 convertase (C4b2a). This bimolecular enzymatic complex is similar to the AP C3 convertase (C3bBb) and converts C3 into C3a and C3b. Due to the feedback or amplification loop, AP activation accounts for most of the activity of the complement system (~80%) even if CP or LP was initially activated. Because the complement system provides a rapidly activated and potent surveillance mechanism for the host, strict control in plasma and on cells is required to avoid damage to self. The fluid-phase regulators are factor H (FH) and C4b-binding protein (C4BP). The membrane-associated regulators are membrane cofactor protein (MCP; CD46), decay-accelerating factor (DAF; CD55) and complement receptor 1 (CR1; CD35). During pregnancy, complement assists in the clearance of placental fragments that enter the maternal circulation as a result of syncytiotrophoblast turn-over [19]. One prevailing hypothesis is that improper clearance of such components, driven by an inadequately regulated complement cascade, may lead to deposition of debris in tissues and vascular walls leading to an overly exuberant inflammatory response [11]. Such unwarranted complement activation at the maternal-foetal border may lead to inadequate spiral artery remodelling, lack of maternal-foetal tolerance and poor placentation, all of which are potential key mechanisms in the pathogenesis of PE [12, 20]. Genetic variants resulting in aberrant protein function in complement receptors CR3 and CR4 have been linked to increased risk of PE [19]. We have recently identified and published rare genetic variants in Factor H that predispose to PE, due to excessive complement activation [21].

We designed a targeted exome sequencing protocol to screen the exomes and splicing areas of selected genes within the complement system in PE patients and controls.

## Materials and methods

In this targeted exomic sequencing study, we combined data from exomic sequencing of 487 (body mass index <30 kg/m^2^) PE mothers and 187 (BMI < 30 kg/m^2^) non-PE control mothers from the Finnish Genetics of Pre-eclampsia Consortium (FINNPEC) [22]. This data was combined with 122 women with a history of PE as additional cases and 1905 parous women with no such history as additional controls from the national FINRISK study (THL Biobank permit no BB2016_8; study design in Supplementary Fig. s1). From the FINNPEC cohort, nulliparous or multiparous women with a singleton pregnancy and no history of chronic hypertension, diabetes or renal disease were included, 92.4% of cases and 100% of controls were primiparous and the cases and controls did not differ in age or BMI. All patients were of European ancestry. A jury consisting of a midwife and an obstetrician independently confirmed the diagnosis of PE. For the diagnosis, newly-onset hypertension and proteinuria after 20 weeks of gestation were required. Hypertension was defined as a systolic blood pressure of ≥140 mm Hg and/or a diastolic blood pressure of ≥90 mm Hg after 20 weeks of gestation. Proteinuria was defined as the urinary excretion of ≥0.3 g protein in a 24-h specimen or >0.3 g/l of protein in urine, or two positive dipstick readings in the absence of a urinary tract infection. The diagnosis of PE in FINRISK was based on a clinician’s diagnosis obtained through the comprehensive national Hospital Discharge Registry. For genetic association analyses, data from FINNPEC and FINRISK was combined and in the pooled case-control cohort.

In a previously described custom-made targeted exomic sequencing protocol, we combined Illumina sequencing libraries for capture and sequencing with Nimblegen sequence capture [23, 24]. The subjects and methods of this study have been described in detail previously and are explained in the online supplement [19, 21, 24, 25].

### Statistics

Sequence data were analyzed in PLINK/Seq, Plink [26] and R programmes. Quality control before meta-analysis included removal of singleton and monomorphic variants, removal of sites with >10% missing data in the targeted sequencing or a significant departure from Hardy-Weinberg equilibrium in controls (*p* < 0.001). Analyses of the significant associations were performed by the Fisher’s exact test. *P* values < 0.05 were considered to indicate a significant difference. The variants that are considered benign were used to confirm that the overall study does not have unexpected inflation from technical or population structure issues. The tail of the *p* value distribution of benign variants was as expected, suggesting that the overall study design and quality control were successful without multiple test adjustments. In addition to an appropriate statistical probability test, odds ratios (OR) with 95% confidence intervals (CI95) were calculated for all variants.

### Study approval

This research was performed in accordance with the Declaration of Helsinki. All subjects provided a written informed consent for the study. The FINNPEC study protocol was approved by the coordinating Ethics Committee of the Hospital District of Helsinki and Uusimaa (permit number 149/E0/07). National FINRISK study description and ethical approvals are available online: https://thl.fi/documents/155392151/190122053/FINRISK_readmefirst_220920.pdf/aafab52b-a53b-d23c-73d7-15c5581b24fa/FINRISK_readmefirst_220920.pdf?t=1630303397716#:~:text=The%20transfer%20of%20the%20FINRISK,Health%20on%209%20March%202015.

## Results

To analyze if variants in genes coding for 40 components of the complement system are associated with PE, we performed association testing on Finnish preeclamptic mothers (609) and controls (2092). The studied genes are listed in Supplementary table 1 and results of these genetic association analyses are listed in Table 1, Of the 14 associating variants, 11 were rare (MAF < 0.1) and, of these, 6 were missense variants. The lack of inflated *p* values in comparison of benign and putatively functional variants indicates that confounders such as stratification are not causing false positives. In total, 1640 variants were observed (Supplementary Table 2).

**Table 1:** Genetic variants within genes coding for components of the complement system that are associated with preeclampsia (*p* ≤ 0.05).

RSID	Gene name	*P* value	OR (95% confidence interval)	MAF cases/MAF controls (%)	Consequence	ACMG/ClinVar	Role of gene
rs45532534	C3	0.01	13.80 (1.36–677.10)	0.33/0.02	c.4260+39 C > A (ENST00000245907.11)	VUS/NA	AP activation
rs2230201	C3	0.04	1.17 (1.01–1.37)	30.61/26.08	p.Arg304= (ENSP00000245907.4): TF binding site	Benign/Benign	AP activation
rs41258244	CD46	0.05	1.37 (0.995–1.863)	5.15/3.82	c.476-41 A > T (ENST00000367042.6)	VUS/Benign	CP and AP inhibition, surface-bound
rs1800947	CRP	0.02	1.36 (1.06–1.74)	7.66/5.69	p.Leu184= (ENSP00000255030.5)	VUS/NA	CP activation, AP inhibition
rs3828032	CFHR2	0.02	0.83 (0.70–0.97)	26.93/22.22	c.430+20 C > T (ENST00000367415.8)	VUS/Benign	AP regulation
rs114727460	CFHR4	0.03	6.86 (0.98–75.74)	0.33/0.05	c.437-34 T > C (ENST00000367416.6)	VUS/NA	AP regulation
rs7417769	CFHR4	0.01	0.81 (0.69–0.96)	25.62/20.77	p.Asn210Ser (ENSP00000477162.2)	VUS/Benign	AP regulation
rs35662416	CFHR5	0.03	1.82 (1.03–3.13)	1.81/1.00	p.Arg356His (ENSP00000256785.4)	VUS/Likely-Benign	AP regulation
rs200674959	C5	0.01	24.13 (1.25–467.43)	0.25/0	p.Ile1291Val (ENSP00000223642.1)	VUS/VUS	TP activation
rs147430470	C5	0.01	22.75 (1.17–440.78)	0.25/0	p.Ile330Thr (ENSP00000223642.1)	VUS/Likely-Benign	TP activation
rs41271067	C6	0.01	2.93 (1.18–7.10)	0.90/0.31	p.Asp696Gly (ENSP00000338861.5)	VUS/Benign	TP activation
rs114609505	C6	0.02	0.47 (0.22–0.92)	0.82/1.73	p.Thr181Lys (ENSP00000338861.5)	VUS/Likely-Benign	TP activation
rs605648	C8B	0.01	1.34 (1.08–1.66)	11.56/8.62	c.1622-20 A > C (ENST00000371237.9)	VUS/Benign	TP activation
rs72670361	C8B	0.02	1.46 (1.06–2.01)	4.94/3.43	c.1234+40 T > C (ENST00000371237.9)	VUS/NA	TP activation

*RSID* SNP identifier, *OR* odds ratio, *MAF* minor allele frequency in total sample, *VUS* variant of uncertain significance, *NA* not available, *C* complement, *CP* classical pathway; *TP* terminal pathway, *AP* alternative pathway.

**Fig. 1:**
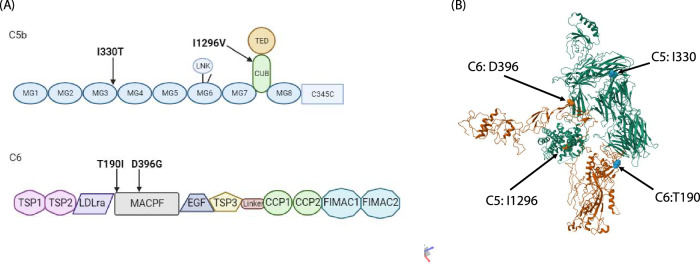
The structure of C5 and C6 proteins. **A** The domain structure of C5b (top) and C6 (bottom) with PE associating variants indicated. The C5 variants are located in exon 31/41 (I330) coding the Immunoglobulin-like domain 3 (MG3) and in exon 9/41 (I1296) coding the conserved complement-activating CUB (complement C1r/C1s, Uegf, Bmp1) domain Both C6 variants in exons 6/16 (T190) and 12/16 (D396) are located in the membrane attack complex/perforin domain (MACPF). **B** The protein structure of C6 (orange) in complex with C5b (green) with the PE-associating amino acids’ positions indicated. The D396 is located directly in the interaction site between the two molecules. The structural model is based on construct 4A5W [37].

Interestingly, two rare missense variants in both *C5* and *C6* were found to associate with PE (Fig. 1A). Of these, the rs200674959 (I1296V), located in conserved complement-activating CUB (complement C1r/C1s, Uegf, Bmp1) domain and rs147430470 (I330T) located in Macroglobulin-like domain 3 (MG3) in *C5*, were predisposing variants (OR = 24.13, CI95 = 1.25–467.43; *p* = 0.01 and OR = 22.75, CI95 = 1.17–440.78; *p* = 0.01, respectively). Also, the rs41271067 (D396G) variant in *C6* was predisposing (OR = 2.93, CI95 = 1.18–7.10; *p* = 0.01), while rs114609505 (T190I) had a protective effect (OR = 0.47, CI95 = 0.22–0.92; *p* = 0.02). Both of the associating *C6* variants were located in the membrane attack complex/perforin domain (MACPF). No associating variants were discovered in the MAC inhibitor protectin (*CD59*).

Other potential associations were found in rare predisposing variants in genes coding for CRP (rs1800947, *p* = 0.02), CFI (rs200040240, *p* = 0.003), C3 (rs45532534, *p* = 0.011) and C5 (rs200674959, *p* = 0.011, discussed above). The strongest protective associations were found in common variants in genes coding for CFHR4 (rs7417769, *p* = 0.012) and C8B (rs605648, *p* = 0.01). To summarize, associating variants were found in genes coding for components of the classical and alternative pathways, but not in the activating components of the lectin pathway. Importantly, six variants were found in the common terminal pathway.

## Discussion

In this study we identified that rare missense variants in genes coding for C5 and C6 of the terminal pathway of complement system activation are associated with PE. We have previously described genetic associations of PE to C3, complement receptors CR3 and CR4 and to the key regulator factor H. Taken together our findings suggest that abnormalities in the alternative and terminal pathway are of particular importance to PE pathogenesis. Potential or known disease associations of the PE-associated variants discovered in this study are scarce in literature.

The terminal pathway of complement activation has received less attention in PE studies than the classical or alternative pathways. CD59 is the only membrane-bound regulator of the terminal pathway, which is attached to the surface of the endothelium and trophoblast cells by a glycophosphatidylinositol (GPI) anchor. The plasma levels of CD59 are increased in PE and associated with end-organ injury related laboratory measures often observed in severe PE, such as elevated liver enzymes and lactase dehydrogenase and decreased platelet count [27]. We have previously shown that CD59 is abundant in the placental trophoblasts, where it is exposed to damage by turbulent intervillous maternal circulation, a characteristic of the PE placenta caused by inadequate uterine arterial remodulation associated with the disease aetiology [18, 28]. It is possible that shear damage caused to the syncytiotrophoblast releases CD59 to maternal circulation thereby leaving the villous tissue vulnerable to aberrant complement attack. Terminal complement complex deposits have been localized in the fibrinoid material of the decidua of the basal plate, in the stroma of the chorionic villi and in the vessel walls, and their levels are increased in preeclamptic placentas [29]. This may be exacerbated in patients with MAC-coding genetic variants. Both associating variants in *C6* are located in the membrane attack complex/perforin domain, which is responsible the MAC function of C6. The D396G is directly in the interaction site between C6 and C5b (Fig.  1B).

We have previously shown that genetic variants of complement component 3 (*C3*) are associated with susceptibility to PE [25]. The protective variant rs2230201 in PE is known to relate to levels of C3 in serum as well as to associate with dense deposit disease (DDD) and systemic lupus erythematosus (SLE) [30–32]. Furthermore, the rs2230201 observed in this study corroborates our previous finding of a *C3* haplotype association to PE susceptibility [25].

CRP is an inflammation marker and trigger of the CP of complement activation. In addition, CRP controls the AP to promote iC3b-mediated clearance of debris by recruiting factor H [33]. The minor PE-associated allele of rs1800947 in *CRP* has been found to be associated with a reduced plasma level of CRP in an elderly Finnish population [34]. We found that the rs1800947 predisposes to PE (*p* value = 0.021; OR 1.356 CI95 = 1.044–1.748). While the variant, also known as CRP2 is synonymous, it has been suggested to have a possible regulatory function. Studies in complex diseases have shown an association of rs1800947, for example, with systemic lupus erythematosus, the severity of prostate cancer and protection from cardiovascular disease [35–37].

This study adds to previous evidence by us and others indicating that in discordant patients, rare complement variants may play an important role in risk for PE. Variants of uncertain significance in genes coding for components of the terminal pathway of complement system may, in the context of physiological changes typical to PE become pathogenic. This is in support of the multiple hit theory of PE aetiology, where multiple factors, one being a rare complement variant, together create the perfect storm of disease-causing conditions.

## Supplementary information

Supplementary materials

## Data Availability

The datasets generated during and/or analysed during the current study are not publicly available due to patient confidentiality but are available from Professor Hannele Laivuori (hannele.laivuori@helsinki.fi) on reasonable request.
