# Supplementary material for: Understanding rare genetic variants within the terminal pathway of complement system in preeclampsia
Source: Genes Immun. 2024 Dec 17;26(1):22–6. doi: 10.1038/s41435-024-00310-6 (PMC11832413; doi:10.1038/s41435-024-00310-6)
Supplement: Supplementary file 1 — Supplementary materials [file 41435_2024_310_MOESM1_ESM.pdf]

**Supplementary materials**

# Understanding rare genetic variants within the terminal pathway of complement system in preeclampsia

Lokki, A. Inkeri; Triebwasser, Michael; Daly, Emma; FINNPEC; Kurki, Mitja I.; Perola, Markus; Auro, Kirsi; Salmon, Jane E.; Anuja, Java; Daly, Mark; Atkinson, John P.; Laivuori, Hannele; Meri, Seppo

## Suppelementary methods

### Targeted Sequencing and Capture Enrichment

Genes coding for key components of the complement system were chosen as targeted genes. Genomic DNA was used to prepare libraries in-house (Washington University School of Medicine) (1). Enzymes were purchased from Enzymatics (Beverly, MA). In short, the ends of sheared genomic DNA fragments were repaired and treated with T4 DNA Polymerase and T4 DNA Polynucleotide Kinase, effectively phosphorylating the 5' hydroxyl group. Taq Polymerase was used to add an adenosine to the 3' position at each end of the DNA fragment. Illumina adapters including an overhanging “T” were ligated onto the DNA fragment. This was followed by bead-based size selection procedure to remove adapter-dimers and fragments below the desired size. By targeting the two ligated universal adapters on each fragment end, a unique index sequence was added using a PCR reaction.

Sequence capture by hybridization was performed according to the manufacturer’s recommendations with modifications (Roche SeqCap Hybridization and Wash Kit No. 05634261001). Longer blocking oligos containing an additional 7 bp inosine segment for promiscuous pairing with different index sequences were used. After hybridization, captured DNA was washed and eluted according to the manufacturer’s instructions.

### Sequencing and Analysis

Sequencing was performed on an Illumina HiSeq 2000 at the Washington University Genome Technology Access Center using 2x101 bp, 2x135 bp and 2x150 bp reads. We aligned sequencing to GRCh37 using bwa

aln (v0.6.1-r104) and genotyped the samples using the Genome Analysis ToolKit (v2.5.2-gf57256b) Unified Genotyper (15-17). The Finrisk cohort samples were genotyped by Agilent 1.1 refseq (60 cases and 4 controls), Illumina coding v1 (162 cases and 10 controls) and Nimblegen SeqCap EZ VCRome (1682 cases and 109 controls) platforms.

**Supplementary references**

1. Triebwasser M. Excessive Complement Activation Due to Genetic Haploinsufficiency of Regulators in Multiple Human Diseases. Washington University in St.Louis, Arts & Sciences Electronic Theses and Dissertations. 2015. Available from: http://openscholarship.wustl.edu/art_sci_etds/427

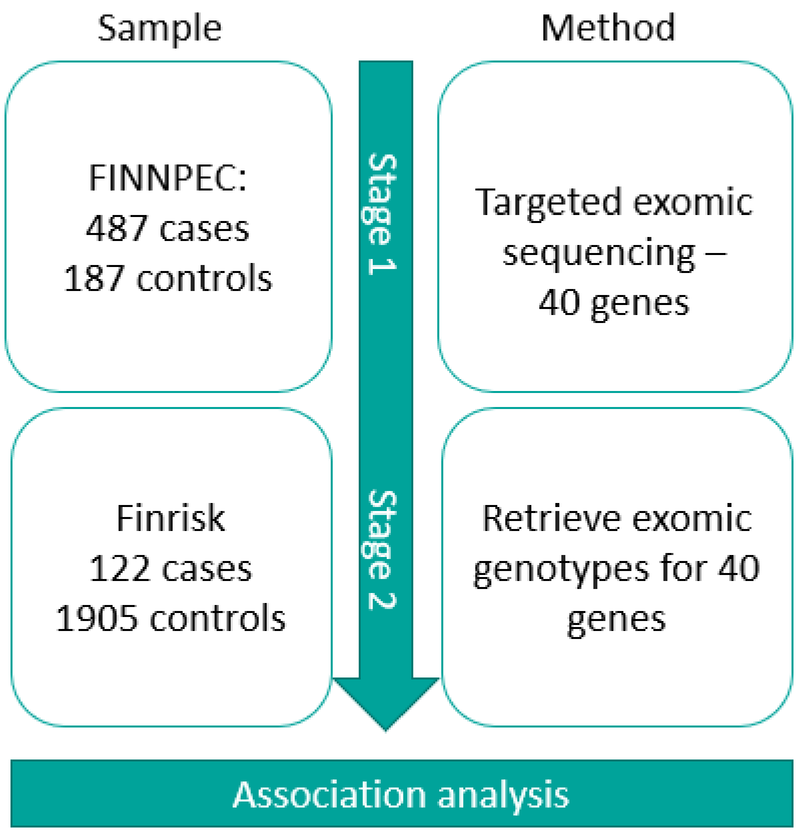


**Supplementary figure S1**. The study design included combination of two sample cohorts, FINNPEC and Finrisk for genetic association analysis.

**Supplementary table 1.** The genes studied in this targeted exomic sequencing study.

AP – alternative pathway, CP – classical pathway, LP- lectin pathway , - TP – terminal pathway

| Gene name | Complement pathway | Complement component |
|---|---|---|
| C1QA | CP | Activator |
| C1QB | CP | Activator |
| C1QBP | CP | Activator |
| C1QC | CP | Activator |
| C1R | CP | Activator |
| C1S | CP | Activator |
| C2 | CP | Activator |
| C3 | CP/AP/LP | Activator |
| C4A | CP/LP | Activator |
| C4BPA | CP/LP | Inhibitor |
| C4BPB | CP/LP | Inhibitor |
| C5 | TP | Activator |
| C6 | TP | Activator |
| C7 | TP | Activator |
| C8A | TP | Activator |
| C8B | TP | Activator |
| C8G | TP | Activator |
| C9 | TP | Activator |
| CD46 | CP/AP/LP | Inhibitor |
| CD55 | CP/AP/LP | Inhibitor |
| CD59 | TP | Inhibitor |
| CFB | AP | Activator |
| CFD | AP | Activator |
| CFHR1 | AP | Regulator |
| CFHR2 | AP | Regulator |
| CFHR3 | AP | Regulator |
| CFHR4 | AP | Regulator |
| CFHR5 | AP | Regulator |
| CFI | CP/AP/LP | Inhibitor |
| CFP | AP | Activator |
| CLU | TP | Inhibitor |
| CRP | CP | Activator |
| MASP1 | LP | Activator |
| MASP2 | LP | Activator |
| MBL2 | LP | Activator |
| SERPINA1 | CP/AP | Inhibitor |
| SERPINB10 | CP/AP | Inhibitor |
| SERPING1 | CP | Inhibitor |
| VTN | TP | Inhibitor |
| CTB-96E2.2 | Uncharacterized | Paralog of VTN |

**Supplementary table 2.** All observed variants in the genes listed in Supplementary table 1. Variant positions are determined according to GRCh37.p13.

| Variant positions | Gene | MAF |
|---|---|---|
| chr1:11086717 | MASP2 | 0.836 |
| chr1:11086723 | MASP2 | 0.001439 |
| chr1:11087432 | MASP2 | 0.0007184 |
| chr1:11087456 | MASP2 | 0.0007184 |
| chr1:11087524 | MASP2 | 0.836 |
| chr1:11087612 | MASP2 | 0.014 |
| chr1:11090374..11090375 | MASP2 | 0.0007194 |
| chr1:11090475 | MASP2 | 0.0007205 |
| chr1:11090897 | MASP2 | 0.01 |
| chr1:11090916 | MASP2 | 0.826 |
| chr1:11091031 | MASP2 | 0.01 |
| chr1:11091097 | MASP2 | 0.002878 |
| chr1:11091106 | MASP2 | 0.0007194 |
| chr1:11091205 | MASP2 | 0.111 |
| chr1:11094638 | MASP2 | 0.001739 |
| chr1:11094665 | MASP2 | 0.826 |
| chr1:11094877 | MASP2 | 0.0007184 |
| chr1:11095039 | MASP2 | 0.837 |
| chr1:11095080 | MASP2 | 0.002158 |
| chr1:11097867 | MASP2 | 0.019 |
| chr1:11102703 | MASP2 | 0.005764 |
| chr1:11102764 | MASP2 | 0.0007205 |
| chr1:11102888 | MASP2 | 0.0007184 |
| chr1:11102940 | MASP2 | 0.017 |
| chr1:11103005 | MASP2 | 0.0007194 |
| chr1:11103300 | MASP2 | 0.002878 |
| chr1:11103326 | MASP2 | 0.0007194 |
| chr1:11103348 | MASP2 | 0.003597 |
| chr1:11103382 | MASP2 | 0.0007184 |
| chr1:11103408 | MASP2 | 0.0007184 |
| chr1:11103784 | MASP2 | 0.0007194 |
| chr1:11104712 | MASP2 | 0.775 |
| chr1:11104845 | MASP2 | 0.8 |
| chr1:11104851 | MASP2 | 0.0007184 |
| chr1:11105106 | MASP2 | 0.782 |
| chr1:11105122 | MASP2 | 0.802 |
| chr1:11105159 | MASP2 | 0.0007184 |
| chr1:11105237 | MASP2 | 0.054 |
| chr1:11105542 | MASP2 | 0.00431 |
| chr1:11105873 | MASP2 | 0.0007386 |
| chr1:11106317 | MASP2 | 0.019 |
| chr1:11106321 | MASP2 | 0.0007184 |
| chr1:11106359 | MASP2 | 0.801 |
| chr1:11106492 | MASP2 | 0.0007184 |

| | | |
|---|---|---|
| chr1:11106617 | MASP2 | 0.0007194 |
| chr1:11106642 | MASP2 | 0.001439 |
| chr1:11106831 | MASP2 | 0.0007184 |
| chr1:11106855 | MASP2 | 0.0007184 |
| chr1:11106909 | MASP2 | 0.01 |
| chr1:11107392 | MASP2 | 0.0007184 |
| chr1:11107439 | MASP2 | 0.759 |
| chr1:22963971 | C1QA | 0.0007194 |
| chr1:22964015 | C1QA | 0.0007194 |
| chr1:22964065 | C1QA | 0.0007184 |
| chr1:22964176 | C1QA | 0.007902 |
| chr1:22964367 | C1QA | 0.002874 |
| chr1:22964407 | C1QA | 0.0007184 |
| chr1:22965277 | C1QA | 0.0007194 |
| chr1:22965416 | C1QA | 0.0007194 |
| chr1:22966045 | C1QA | 0.0007194 |
| chr1:22970292 | C1QC | 0.002158 |
| chr1:22970432 | C1QA | 0.008621 |
| chr1:22970621 | C1QC | 0.0007184 |
| chr1:22970642 | C1QC | 0.208 |
| chr1:22970790 | C1QC | 0.075 |
| chr1:22970797..22970798 | C1QC | 0.085 |
| chr1:22970828 | C1QC | 0.439 |
| chr1:22970883 | C1QC | 0.018 |
| chr1:22970908 | C1QC | 0.075 |
| chr1:22970925 | C1QC | 0.368 |
| chr1:22973699 | C1QC | 0.047 |
| chr1:22973802 | C1QC | 0.0007184 |
| chr1:22973979 | C1QC | 0.0007194 |
| chr1:22973996 | C1QC | 0.001439 |
| chr1:22974297 | C1QC | 0.368 |
| chr1:22974549 | C1QC | 0.033 |
| chr1:22985870 | C1QB | 0.0007194 |
| chr1:22986398 | C1QB | 0.0007257 |
| chr1:22986403 | C1QB | 0.103 |
| chr1:22987070 | C1QB | 0.041 |
| chr1:22987099 | C1QB | 0.002158 |
| chr1:22987147 | C1QB | 0.008633 |
| chr1:22987511 | C1QB | 0.001437 |
| chr1:22987720 | C1QB | 0.0007184 |
| chr1:22987958 | C1QB | 0.968 |
| chr1:22988029 | C1QB | 0.0007184 |
| chr1:22988076 | C1QB | 0.0007205 |
| chr1:57320317 | C8A | 0.005102 |
| chr1:57320444 | C8A | 0.052 |
| chr1:57320487 | C8A | 0.002878 |
| chr1:57333292 | C8A | 0.0007194 |
| chr1:57333311 | C8A | 0.002878 |
| chr1:57333312 | C8A | 0.004317 |
| chr1:57340333 | C8A | 0.109 |
| chr1:57340381 | C8A | 0.186 |

| | | |
|---|---|---|
| chr1:57340475 | C8A | 0.156 |
| chr1:57340546 | C8A | 0.083 |
| chr1:57340727 | C8A | 0.348 |
| chr1:57340860 | C8A | 0.265 |
| chr1:57341602 | C8A | 0.01 |
| chr1:57341625 | C8A | 0.082 |
| chr1:57341803 | C8A | 0.0007194 |
| chr1:57341893 | C8A | 0.0007184 |
| chr1:57342080 | C8A | 0.0007194 |
| chr1:57342178 | C8A | 0.005087 |
| chr1:57346834 | C8A | 0.005277 |
| chr1:57346990 | C8A | 0.007914 |
| chr1:57347171 | C8A | 0.0007184 |
| chr1:57347244 | C8A | 0.002874 |
| chr1:57347259 | C8A | 0.0007184 |
| chr1:57347323 | C8A | 0.0007184 |
| chr1:57347443 | C8A | 0.007902 |
| chr1:57349079 | C8A | 0.002177 |
| chr1:57349407 | C8A | 0.18 |
| chr1:57349464 | C8A | 0.181 |
| chr1:57349541 | C8A | 0.001443 |
| chr1:57349583..57349584 | C8A | 0.188 |
| chr1:57349614 | C8A | 0.335 |
| chr1:57351445 | C8A | 0.148 |
| chr1:57351488 | C8A | 0.0007205 |
| chr1:57351943 | C8A | 0.024 |
| chr1:57372162..57372163 | C8A | 0.015 |
| chr1:57372591 | C8A | 0.763 |
| chr1:57372687 | C8A | 0.002963 |
| chr1:57373500..57373503 | C8A | 0.243 |
| chr1:57373626 | C8A | 0.0007184 |
| chr1:57373737 | C8A | 0.008621 |
| chr1:57374000 | C8A | 0.037 |
| chr1:57377779 | C8A | 0.037 |
| chr1:57377810 | C8A | 0.892 |
| chr1:57377886 | C8A | 0.039 |
| chr1:57378149 | C8A | 0.038 |
| chr1:57378150 | C8A | 0.038 |
| chr1:57378198 | C8A | 0.038 |
| chr1:57378255 | C8A | 0.0007184 |
| chr1:57378334 | C8A | 0.038 |
| chr1:57378395 | C8A | 0.0007184 |
| chr1:57378413 | C8A | 0.899 |
| chr1:57378523 | C8A | 0.0007236 |
| chr1:57383315 | C8A | 0.038 |
| chr1:57383337 | C8A | 0.0007194 |
| chr1:57383353 | C8A | 0.001439 |
| chr1:57383357 | C8A | 0.004317 |
| chr1:57383358 | C8A | 0.116 |
| chr1:57383376 | C8A | 0.0007194 |
| chr1:57383638..57383639 | C8A | 0.017 |

| | | |
|---|---|---|
| chr1:57383683 | C8A | 0.036 |
| chr1:57394993 | C8B | 0.002874 |
| chr1:57395020 | C8B | 0.398 |
| chr1:57395053 | C8B | 0.0007184 |
| chr1:57395067 | C8B | 0.0007184 |
| chr1:57395137 | C8B | 0.0007184 |
| chr1:57395228 | C8B | 0.002874 |
| chr1:57395251 | C8B | 0.9 |
| chr1:57395294 | C8B | 0.051 |
| chr1:57395321 | C8B | 0.025 |
| chr1:57395421 | C8B | 0.398 |
| chr1:57395434 | C8B | 0.128 |
| chr1:57397183 | C8B | 0.41 |
| chr1:57397301 | C8B | 0.396 |
| chr1:57397497 | C8B | 0.0007184 |
| chr1:57397733 | C8B | 0.025 |
| chr1:57397738 | C8B | 0.399 |
| chr1:57398793 | C8B | 0.001439 |
| chr1:57398949 | C8B | 0.003592 |
| chr1:57398952 | C8B | 0.0007184 |
| chr1:57399250 | C8B | 0.025 |
| chr1:57399324 | C8B | 0.0007205 |
| chr1:57406392 | C8B | 0.476 |
| chr1:57406582 | C8B | 0.0007194 |
| chr1:57406638 | C8B | 0.001437 |
| chr1:57406866 | C8B | 0.025 |
| chr1:57406927 | C8B | 0.403 |
| chr1:57409112 | C8B | 0.002928 |
| chr1:57409204 | C8B | 0.009353 |
| chr1:57409210 | C8B | 0.0007194 |
| chr1:57409327 | C8B | 0.041 |
| chr1:57409329 | C8B | 0.047 |
| chr1:57409459 | C8B | 0.005755 |
| chr1:57409473 | C8B | 0.0007194 |
| chr1:57409744 | C8B | 0.052 |
| chr1:57411478 | C8B | 0.0007184 |
| chr1:57411596 | C8B | 0.0007184 |
| chr1:57411779 | C8B | 0.047 |
| chr1:57415200 | C8B | 0.054 |
| chr1:57415310 | C8B | 0.043 |
| chr1:57415594 | C8B | 0.688 |
| chr1:57415595 | C8B | 0.055 |
| chr1:57415621 | C8B | 0.0007205 |
| chr1:57415723 | C8B | 0.203 |
| chr1:57417698 | C8B | 0.002878 |
| chr1:57417781 | C8B | 0.0007194 |
| chr1:57417923 | C8B | 0.002878 |
| chr1:57417930 | C8B | 0.0007194 |
| chr1:57418056 | C8B | 0.035 |
| chr1:57418132 | C8B | 0.0007215 |
| chr1:57418133 | C8B | 0.001443 |

| | | |
|---|---|---|
| chr1:57420059 | C8B | 0.000731 |
| chr1:57420100 | C8B | 0.0007215 |
| chr1:57420158 | C8B | 0.002155 |
| chr1:57420330 | C8B | 0.0007194 |
| chr1:57420512 | C8B | 0.01 |
| chr1:57420590 | C8B | 0.0007194 |
| chr1:57420627 | C8B | 0.043 |
| chr1:57420764 | C8B | 0.003618 |
| chr1:57422218 | C8B | 0.688 |
| chr1:57422298 | C8B | 0.001439 |
| chr1:57422484 | C8B | 0.978 |
| chr1:57422511 | C8B | 0.043 |
| chr1:57422819 | C8B | 0.002161 |
| chr1:57425290 | C8B | 0.001439 |
| chr1:57431546 | C8B | 0.0007184 |
| chr1:57431629 | C8B | 0.019 |
| chr1:57431827 | C8B | 0.024 |
| chr1:57431890 | C8B | 0.01 |
| chr1:159683041 | CRP | 0.002177 |
| chr1:159683091 | CRP | 0.358 |
| chr1:159683099 | CRP | 0.0007194 |
| chr1:159683438 | CRP | 0.075 |
| chr1:159683686 | CRP | 0.0007194 |
| chr1:159683814 | CRP | 0.001439 |
| chr1:159684186 | CRP | 0.356 |
| chr1:173876561 | SERPINC1 | 0.102 |
| chr1:173876705 | SERPINC1 | 0.111 |
| chr1:173876913 | SERPINC1 | 0.0007564 |
| chr1:173878403 | SERPINC1 | 0.00365 |
| chr1:173878471 | SERPINC1 | 0.269 |
| chr1:173878528 | SERPINC1 | 0.001441 |
| chr1:173878832 | SERPINC1 | 0.309 |
| chr1:173878862 | SERPINC1 | 0.293 |
| chr1:173878970 | SERPINC1 | 0.003592 |
| chr1:173878972 | SERPINC1 | 0.0007184 |
| chr1:173879207 | SERPINC1 | 0.024 |
| chr1:173879782 | SERPINC1 | 0.004317 |
| chr1:173880752 | SERPINC1 | 0.082 |
| chr1:173880779 | SERPINC1 | 0.002874 |
| chr1:173883428 | SERPINC1 | 0.005178 |
| chr1:173883517 | SERPINC1 | 0.083 |
| chr1:173883881 | SERPINC1 | 0.005755 |
| chr1:173883970 | SERPINC1 | 0.0007194 |
| chr1:173883984 | SERPINC1 | 0.0007194 |
| chr1:173884010 | SERPINC1 | 0.002874 |
| chr1:173886216 | SERPINC1 | 0.11 |
| chr1:173886316 | SERPINC1 | 0.001437 |
| chr1:173886519 | SERPINC1 | 0.0007194 |
| chr1:173886626 | SERPINC1 | 0.022 |
| chr1:196743814 | CFHR3 | 0.001464 |
| chr1:196743826 | CFHR3 | 0.185 |

| | | |
|---|---|---|
| chr1:196743964 | CFHR3 | 0.184 |
| chr1:196744331 | CFHR3 | 0.026 |
| chr1:196748363 | CFHR3 | 0.001456 |
| chr1:196748676 | CFHR3 | 0.206 |
| chr1:196748844 | CFHR3 | 0.005474 |
| chr1:196749234 | CFHR3 | 0.001453 |
| chr1:196757298 | CFHR3 | 0.0007289 |
| chr1:196757530 | CFHR3 | 0.002911 |
| chr1:196757728 | CFHR3 | 0.027 |
| chr1:196757859 | CFHR3 | 0.0007215 |
| chr1:196757960 | CFHR3 | 0.023 |
| chr1:196758148 | CFHR3 | 0.187 |
| chr1:196758214 | CFHR3 | 0.193 |
| chr1:196762383 | CFHR3 | 0.005119 |
| chr1:196762784 | CFHR3 | 0.00687 |
| chr1:196762897 | CFHR3 | 0.023 |
| chr1:196789161 | CFHR1 | 0.138 |
| chr1:196789200 | CFHR1 | 0.261 |
| chr1:196794449 | CFHR1 | 0.289 |
| chr1:196795014 | CFHR1 | 0.386 |
| chr1:196795018 | CFHR1 | 0.297 |
| chr1:196795725 | CFHR1 | 0.002868 |
| chr1:196795741 | CFHR1 | 0.388 |
| chr1:196795761 | CFHR1 | 0.381 |
| chr1:196795918 | CFHR1 | 0.012 |
| chr1:196796184 | CFHR1 | 0.384 |
| chr1:196796240 | CFHR1 | 0.389 |
| chr1:196796361 | CFHR1 | 0.388 |
| chr1:196796924 | CFHR1 | 0.001279 |
| chr1:196797204 | CFHR1 | 0.0007321 |
| chr1:196797676 | CFHR1 | 0.026 |
| chr1:196799433 | CFHR1 | 0.377 |
| chr1:196799485 | CFHR1 | 0.002276 |
| chr1:196799542 | CFHR1 | 0.001471 |
| chr1:196799546 | CFHR1 | 0.0007331 |
| chr1:196799643 | CFHR1 | 0.0007289 |
| chr1:196799782 | CFHR1 | 0.0007278 |
| chr1:196799813 | CFHR1 | 0.0007267 |
| chr1:196800023 | CFHR1 | 0.037 |
| chr1:196800845 | CFHR1 | 0.0007353 |
| chr1:196800889 | CFHR1 | 0.001468 |
| chr1:196801025 | CFHR1 | 0.0007299 |
| chr1:196857150 | CFHR4 | 0.007194 |
| chr1:196871317 | CFHR4 | 0.001464 |
| chr1:196871717 | CFHR4 | 0.003597 |
| chr1:196874192 | CFHR4 | 0.015 |
| chr1:196874356 | CFHR4 | 1 |
| chr1:196875846 | CFHR4 | 0.001449 |
| chr1:196875957 | CFHR4 | 0.002878 |
| chr1:196875968 | CFHR4 | 0.0007194 |
| chr1:196876152 | CFHR4 | 0.0007184 |

| | | |
|---|---|---|
| chr1:196876309..196876310 | CFHR4 | 0.028 |
| chr1:196876395 | CFHR4 | 0.0007215 |
| chr1:196876434 | CFHR4 | 0.025 |
| chr1:196876458 | CFHR4 | 0.791 |
| chr1:196876534 | CFHR4 | 0.0007184 |
| chr1:196876598 | CFHR4 | 0.026 |
| chr1:196876758 | CFHR4 | 0.012 |
| chr1:196876879 | CFHR4 | 0.09 |
| chr1:196876885 | CFHR4 | 0.002257 |
| chr1:196881600 | CFHR4 | 0.494 |
| chr1:196881988 | CFHR4 | 0.002874 |
| chr1:196882009 | CFHR4 | 0.0007184 |
| chr1:196882038 | CFHR4 | 0.001437 |
| chr1:196882243 | CFHR4 | 0.012 |
| chr1:196882344 | CFHR4 | 0.078 |
| chr1:196883372 | CFHR4 | 0.141 |
| chr1:196883469 | CFHR4 | 0.078 |
| chr1:196883807 | CFHR4 | 0.002878 |
| chr1:196883880 | CFHR4 | 0.007968 |
| chr1:196884074 | CFHR4 | 0.147 |
| chr1:196887181 | CFHR4 | 0.224 |
| chr1:196887213 | CFHR4 | 0.018 |
| chr1:196887274 | CFHR4 | 0.127 |
| chr1:196887457 | CFHR4 | 0.143 |
| chr1:196887681 | CFHR4 | 0.0007342 |
| chr1:196887813 | CFHR4 | 0.013 |
| chr1:196918341 | CFHR2 | 0.004425 |
| chr1:196918405 | CFHR2 | 0.001515 |
| chr1:196918738 | CFHR2 | 0.003597 |
| chr1:196918741 | CFHR2 | 0.03 |
| chr1:196919759 | CFHR2 | 0.0008576 |
| chr1:196919989 | CFHR2 | 0.0007194 |
| chr1:196920148 | CFHR2 | 0.286 |
| chr1:196920151 | CFHR2 | 0.024 |
| chr1:196920178 | CFHR2 | 0.219 |
| chr1:196926956 | CFHR2 | 0.219 |
| chr1:196926973 | CFHR2 | 0.219 |
| chr1:196927158 | CFHR2 | 0.0007194 |
| chr1:196927185 | CFHR2 | 0.03 |
| chr1:196927275 | CFHR2 | 0.0007205 |
| chr1:196927428 | CFHR2 | 0.536 |
| chr1:196927771 | CFHR2 | 0.224 |
| chr1:196927788 | CFHR2 | 0.215 |
| chr1:196927791 | CFHR2 | 0.448 |
| chr1:196928188 | CFHR2 | 0.012 |
| chr1:196946546 | CFHR5 | 0.072 |
| chr1:196946641 | CFHR5 | 0.0007194 |
| chr1:196946775 | CFHR5 | 0.065 |
| chr1:196946869 | CFHR5 | 0.229 |
| chr1:196947030 | CFHR5 | 0.308 |
| chr1:196947139 | CFHR5 | 0.062 |

| | | |
|---|---|---|
| chr1:196951912 | CFHR5 | 0.002886 |
| chr1:196951925 | CFHR5 | 0.016 |
| chr1:196952092 | CFHR5 | 0.001439 |
| chr1:196952268 | CFHR5 | 0.227 |
| chr1:196952498 | CFHR5 | 0.269 |
| chr1:196952908 | CFHR5 | 0.14 |
| chr1:196952917 | CFHR5 | 0.032 |
| chr1:196953086 | CFHR5 | 0.006475 |
| chr1:196953159 | CFHR5 | 0.001439 |
| chr1:196953221 | CFHR5 | 0.0007184 |
| chr1:196962982 | CFHR5 | 0.961 |
| chr1:196963006 | CFHR5 | 0.481 |
| chr1:196963055 | CFHR5 | 0.00444 |
| chr1:196963211 | CFHR5 | 0.001441 |
| chr1:196964844 | CFHR5 | 0.0007194 |
| chr1:196964861 | CFHR5 | 0.002158 |
| chr1:196964971 | CFHR5 | 0.012 |
| chr1:196965062 | CFHR5 | 0.004335 |
| chr1:196965193 | CFHR5 | 0.022 |
| chr1:196965448 | CFHR5 | 0.005216 |
| chr1:196967354 | CFHR5 | 0.019 |
| chr1:196967458 | CFHR5 | 0.23 |
| chr1:196967472 | CFHR5 | 0.0007246 |
| chr1:196971432 | CFHR5 | 0.001754 |
| chr1:196973533 | CFHR5 | 0.003604 |
| chr1:196973551 | CFHR5 | 0.00423 |
| chr1:196973582 | CFHR5 | 0.017 |
| chr1:196977644 | CFHR5 | 0.003602 |
| chr1:207262791 | C4BPB | 0.0007174 |
| chr1:207262980 | C4BPB | 0.541 |
| chr1:207263539 | C4BPB | 0.018 |
| chr1:207263829 | C4BPB | 0.524 |
| chr1:207263877 | C4BPB | 0.0007184 |
| chr1:207263927 | C4BPB | 0.0007194 |
| chr1:207263943 | C4BPB | 0.019 |
| chr1:207264715 | C4BPB | 0.005155 |
| chr1:207265205 | C4BPB | 0.005755 |
| chr1:207268612 | C4BPB | 0.0007184 |
| chr1:207268665 | C4BPB | 0.002878 |
| chr1:207268779 | C4BPB | 0.515 |
| chr1:207268860 | C4BPB | 0.52 |
| chr1:207269024 | C4BPB | 0.035 |
| chr1:207269098 | C4BPB | 0.002158 |
| chr1:207269205 | C4BPB | 0.058 |
| chr1:207269854 | C4BPB | 0.0007184 |
| chr1:207269858 | C4BPB | 0.062 |
| chr1:207269919 | C4BPB | 0.134 |
| chr1:207271303 | C4BPB | 0.0007194 |
| chr1:207271350 | C4BPB | 0.001439 |
| chr1:207271387 | C4BPB | 0.0007194 |
| chr1:207271510 | C4BPB | 0.0007184 |

| | | |
|---|---|---|
| chr1:207271862 | C4BPB | 0.043 |
| chr1:207272951 | C4BPA | 0.0007257 |
| chr1:207273311 | C4BPB | 0.0007194 |
| chr1:207273331 | C4BPB | 0.002158 |
| chr1:207273364 | C4BPA | 0.0007194 |
| chr1:207273492 | C4BPA | 0.066 |
| chr1:207286380 | C4BPA | 0.0007184 |
| chr1:207286381 | C4BPA | 0.026 |
| chr1:207286409 | C4BPA | 0.0007184 |
| chr1:207286671 | C4BPA | 0.0007194 |
| chr1:207286757 | C4BPA | 0.079 |
| chr1:207287187 | C4BPA | 0.122 |
| chr1:207287418 | C4BPA | 0.0007194 |
| chr1:207288580 | C4BPA | 0.001445 |
| chr1:207288897 | C4BPA | 0.593 |
| chr1:207288923 | C4BPA | 0.0007246 |
| chr1:207289011 | C4BPA | 0.0007246 |
| chr1:207297484 | C4BPA | 0.379 |
| chr1:207297602 | C4BPA | 0.0007184 |
| chr1:207297622 | C4BPA | 0.0007184 |
| chr1:207297680 | C4BPA | 0.883 |
| chr1:207300070 | C4BPA | 0.065 |
| chr1:207300259 | C4BPA | 0.449 |
| chr1:207300269 | C4BPA | 0.001435 |
| chr1:207300321 | C4BPA | 0.0007184 |
| chr1:207300381 | C4BPA | 0.361 |
| chr1:207300463 | C4BPA | 0.002196 |
| chr1:207304848 | C4BPA | 0.001439 |
| chr1:207304900 | C4BPA | 0.451 |
| chr1:207304980 | C4BPA | 0.006466 |
| chr1:207305097 | C4BPA | 0.001439 |
| chr1:207307590 | C4BPA | 0.0007184 |
| chr1:207307780 | C4BPA | 0.0007194 |
| chr1:207307932 | C4BPA | 0.001439 |
| chr1:207314356 | C4BPA | 0.024 |
| chr1:207314360 | C4BPA | 0.0007184 |
| chr1:207314388 | C4BPA | 0.178 |
| chr1:207314540 | C4BPA | 0.0007194 |
| chr1:207314786 | C4BPA | 0.0007194 |
| chr1:207316953 | C4BPA | 0.025 |
| chr1:207317139 | C4BPA | 0.021 |
| chr1:207495427 | CD55 | 0.46 |
| chr1:207495552 | CD55 | 0.0007194 |
| chr1:207495871 | CD55 | 0.003597 |
| chr1:207495978 | CD55 | 0.003597 |
| chr1:207496029 | CD55 | 0.012 |
| chr1:207496129 | CD55 | 0.276 |
| chr1:207497788 | CD55 | 0.0007246 |
| chr1:207497811 | CD55 | 0.024 |
| chr1:207497911 | CD55 | 0.0007194 |
| chr1:207498185 | CD55 | 0.052 |

| | | |
|---|---|---|
| chr1:207498325 | CD55 | 0.007435 |
| chr1:207498912 | CD55 | 0.002165 |
| chr1:207499086 | CD55 | 0.001439 |
| chr1:207499264 | CD55 | 0.0007225 |
| chr1:207499275 | CD55 | 0.058 |
| chr1:207499365 | CD55 | 0.777 |
| chr1:207499946 | CD55 | 0.001493 |
| chr1:207500032 | CD55 | 0.025 |
| chr1:207500293 | CD55 | 0.756 |
| chr1:207504467 | CD55 | 0.007914 |
| chr1:207504748 | CD55 | 0.746 |
| chr1:207509799 | CD55 | 0.238 |
| chr1:207509937 | CD55 | 0.0007194 |
| chr1:207510289 | CD55 | 1 |
| chr1:207510515 | CD55 | 0.032 |
| chr1:207510548 | CD55 | 0.76 |
| chr1:207510596 | CD55 | 0.759 |
| chr1:207510860 | CD55 | 0.056 |
| chr1:207510905 | CD55 | 0.014 |
| chr1:207512577 | CD55 | 0.999 |
| chr1:207512650 | CD55 | 0.004335 |
| chr1:207527285 | CD55 | 0.303 |
| chr1:207532654 | CD55 | 0.999 |
| chr1:207532841 | CD55 | 0.0008711 |
| chr1:207532883 | CD55 | 0.011 |
| chr1:207532992 | CD55 | 0.0007194 |
| chr1:207533221 | CD55 | 0.0007205 |
| chr1:207925192 | CD46 | 0.583 |
| chr1:207925222 | CD46 | 0.0007194 |
| chr1:207925233 | CD46 | 0.002158 |
| chr1:207925321 | CD46 | 0.0007194 |
| chr1:207925661 | CD46 | 0.002158 |
| chr1:207925932 | CD46 | 0.012 |
| chr1:207930167 | CD46 | 0.018 |
| chr1:207930203 | CD46 | 0.788 |
| chr1:207930459 | CD46 | 0.002878 |
| chr1:207931175 | CD46 | 0.999 |
| chr1:207933011 | CD46 | 0.007225 |
| chr1:207933246 | CD46 | 0.003794 |
| chr1:207933318 | CD46 | 0.597 |
| chr1:207933353 | CD46 | 0.051 |
| chr1:207934553 | CD46 | 0.05 |
| chr1:207934849 | CD46 | 0.213 |
| chr1:207934893 | CD46 | 0.0007205 |
| chr1:207935006 | CD46 | 0.000731 |
| chr1:207940067 | CD46 | 0.033 |
| chr1:207940560 | CD46 | 0.001439 |
| chr1:207940774 | CD46 | 0.0007289 |
| chr1:207940853 | CD46 | 0.153 |
| chr1:207941191 | CD46 | 0.776 |
| chr1:207943797 | CD46 | 0.002404 |

| | | |
|---|---|---|
| chr1:207956559 | CD46 | 0.396 |
| chr1:207956765 | CD46 | 0.0007184 |
| chr1:207956795 | CD46 | 0.019 |
| chr1:207958446 | CD46 | 0.055 |
| chr1:207958611 | CD46 | 0.001468 |
| chr1:207959070 | CD46 | 0.212 |
| chr1:207959659 | CD46 | 0.052 |
| chr1:207959665 | CD46 | 0.586 |
| chr1:207959683 | CD46 | 0.0007225 |
| chr1:207963561 | CD46 | 0.0007194 |
| chr1:207963618 | CD46 | 0.001437 |
| chr1:207966716 | CD46 | 0.02 |
| chr1:207967178 | CD46 | 0.0007257 |
| chr1:207967935 | CD46 | 0.001447 |
| chr3:186937568 | MASP1 | 0.636 |
| chr3:186938234 | MASP1 | 0.024 |
| chr3:186938635 | MASP1 | 0.023 |
| chr3:186938818 | MASP1 | 0.0007184 |
| chr3:186938956 | MASP1 | 0.249 |
| chr3:186939191 | MASP1 | 0.258 |
| chr3:186940797 | MASP1 | 0.068 |
| chr3:186940797..186940798 | MASP1 | 0.0007194 |
| chr3:186941230 | MASP1 | 0.0007205 |
| chr3:186942816 | MASP1 | 0.015 |
| chr3:186943475 | MASP1 | 0.0007194 |
| chr3:186944448 | MASP1 | 0.001439 |
| chr3:186944490 | MASP1 | 0.049 |
| chr3:186947281 | MASP1 | 0.075 |
| chr3:186947410 | MASP1 | 1 |
| chr3:186947411 | MASP1 | 0.042 |
| chr3:186947483 | MASP1 | 0.002155 |
| chr3:186947801 | MASP1 | 0.345 |
| chr3:186947874 | MASP1 | 0.337 |
| chr3:186953226 | MASP1 | 0.709 |
| chr3:186953244 | MASP1 | 0.007914 |
| chr3:186953249 | MASP1 | 0.0007194 |
| chr3:186953321 | MASP1 | 0.188 |
| chr3:186953454 | MASP1 | 0.0007184 |
| chr3:186953477 | MASP1 | 0.0007184 |
| chr3:186953808 | MASP1 | 0.352 |
| chr3:186953835 | MASP1 | 0.0007184 |
| chr3:186953913 | MASP1 | 0.001439 |
| chr3:186953932 | MASP1 | 0.037 |
| chr3:186954138 | MASP1 | 0.001439 |
| chr3:186954139 | MASP1 | 0.001439 |
| chr3:186954264 | MASP1 | 0.0007184 |
| chr3:186954324 | MASP1 | 0.116 |
| chr3:186954545 | MASP1 | 0.005764 |
| chr3:186958968 | MASP1 | 0.369 |
| chr3:186959042 | MASP1 | 0.487 |
| chr3:186959295 | MASP1 | 0.024 |

| | | |
|---|---|---|
| chr3:186961584 | MASP1 | 0.023 |
| chr3:186965032 | MASP1 | 0.0007184 |
| chr3:186965268 | MASP1 | 0.281 |
| chr3:186968067 | MASP1 | 0.017 |
| chr3:186968126 | MASP1 | 0.0007184 |
| chr3:186969250 | MASP1 | 0.0007184 |
| chr3:186969371 | MASP1 | 0.28 |
| chr3:186969390 | MASP1 | 0.022 |
| chr3:186969489 | MASP1 | 0.0007194 |
| chr3:186969554 | MASP1 | 0.0007184 |
| chr3:186969619 | MASP1 | 0.0007184 |
| chr3:186969833 | MASP1 | 0.023 |
| chr3:186970697 | MASP1 | 0.14 |
| chr3:186970808 | MASP1 | 0.756 |
| chr3:186970838 | MASP1 | 0.0007184 |
| chr3:186971296 | MASP1 | 0.285 |
| chr3:186974155 | MASP1 | 0.114 |
| chr3:186974465 | MASP1 | 0.0007184 |
| chr3:186974548 | MASP1 | 0.0007184 |
| chr3:186974803 | MASP1 | 0.022 |
| chr3:186978255 | MASP1 | 0.022 |
| chr3:186978283 | MASP1 | 1 |
| chr3:186978284 | MASP1 | 0.373 |
| chr3:186978515 | MASP1 | 0.012 |
| chr3:186978571 | MASP1 | 0.0007194 |
| chr3:186980264 | MASP1 | 0.171 |
| chr3:186980439 | MASP1 | 0.004317 |
| chr3:186980440 | MASP1 | 0.0007194 |
| chr3:187003450 | MASP1 | 0.002155 |
| chr3:187003495 | MASP1 | 0.877 |
| chr3:187003957 | MASP1 | 0.0007194 |
| chr3:187009176 | MASP1 | 0.077 |
| chr3:187009351 | MASP1 | 0.124 |
| chr3:187009372 | MASP1 | 0.124 |
| chr3:187009390 | MASP1 | 0.001437 |
| chr3:187009412 | MASP1 | 0.124 |
| chr3:187009486 | MASP1 | 0.026 |
| chr3:187009494 | MASP1 | 0.0007184 |
| chr3:187009702 | MASP1 | 0.0007184 |
| chr3:187009738 | MASP1 | 0.125 |
| chr4:110661869 | CFI | 0.007491 |
| chr4:110661907 | CFI | 0.000768 |
| chr4:110661937 | CFI | 0.999 |
| chr4:110662220 | CFI | 0.0007184 |
| chr4:110662247 | CFI | 0.0007184 |
| chr4:110663642 | CFI | 0.002869 |
| chr4:110663770 | CFI | 0.0007184 |
| chr4:110663864 | CFI | 0.137 |
| chr4:110663997 | CFI | 1 |
| chr4:110667184 | CFI | 0.015 |
| chr4:110667345 | CFI | 0.027 |

| | | |
|---|---|---|
| chr4:110667421 | CFI | 0.0007194 |
| chr4:110667460 | CFI | 0.0007194 |
| chr4:110667590 | CFI | 0.024 |
| chr4:110667819 | CFI | 0.0007342 |
| chr4:110670339 | CFI | 0.001437 |
| chr4:110670758 | CFI | 0.0007205 |
| chr4:110670807 | CFI | 1 |
| chr4:110670903 | CFI | 0.025 |
| chr4:110673500 | CFI | 0.000744 |
| chr4:110673506 | CFI | 0.002226 |
| chr4:110673739 | CFI | 0.0007257 |
| chr4:110673762 | CFI | 0.00218 |
| chr4:110673883 | CFI | 0.0008 |
| chr4:110673894 | CFI | 0.0008104 |
| chr4:110673953 | CFI | 1 |
| chr4:110678819..110678820 | CFI | 0.285 |
| chr4:110678925 | CFI | 0.999 |
| chr4:110679002 | CFI | 1 |
| chr4:110679378 | CFI | 1 |
| chr4:110681136 | CFI | 0.057 |
| chr4:110681505 | CFI | 0.283 |
| chr4:110681527 | CFI | 0.0007184 |
| chr4:110681618 | CFI | 1 |
| chr4:110681679 | CFI | 0.0007194 |
| chr4:110681994 | CFI | 0.001458 |
| chr4:110682487 | CFI | 0.618 |
| chr4:110682643 | CFI | 0.002874 |
| chr4:110682953 | CFI | 0.259 |
| chr4:110683138 | CFI | 0.986 |
| chr4:110685407 | CFI | 0.005232 |
| chr4:110685623 | CFI | 0.0007194 |
| chr4:110685686 | CFI | 0.0007194 |
| chr4:110685687 | CFI | 0.0007194 |
| chr4:110685820 | CFI | 0.0007194 |
| chr4:110685962 | CFI | 0.027 |
| chr4:110686001 | CFI | 0.999 |
| chr4:110686054 | CFI | 1 |
| chr4:110686079 | CFI | 0.022 |
| chr4:110686084 | CFI | 0.999 |
| chr4:110687719 | CFI | 0.002158 |
| chr4:110687723 | CFI | 0.0007194 |
| chr4:110688119 | CFI | 0.024 |
| chr4:110722886 | CFI | 0.749 |
| chr4:110723140 | CFI | 0.016 |
| chr5:39285139 | C9 | 0.901 |
| chr5:39285172 | C9 | 0.0007215 |
| chr5:39285399 | C9 | 0.0007194 |
| chr5:39285405 | C9 | 0.0007194 |
| chr5:39288820 | C9 | 0.0007194 |
| chr5:39289043 | C9 | 0.003608 |
| chr5:39289046 | C9 | 0.0007205 |

| | | |
|---|---|---|
| chr5:39306515 | C9 | 0.032 |
| chr5:39306604 | C9 | 0.005029 |
| chr5:39306622 | C9 | 0.0007184 |
| chr5:39307128 | C9 | 0.583 |
| chr5:39307131 | C9 | 0.59 |
| chr5:39308330 | C9 | 0.002874 |
| chr5:39308498 | C9 | 0.0007184 |
| chr5:39308735 | C9 | 0.001565 |
| chr5:39311112 | C9 | 0.0007194 |
| chr5:39311121 | C9 | 0.0007194 |
| chr5:39311285 | C9 | 0.0007194 |
| chr5:39311336 | C9 | 0.0007194 |
| chr5:39311421 | C9 | 0.005036 |
| chr5:39315745 | C9 | 0.4 |
| chr5:39315912 | C9 | 0.003597 |
| chr5:39316095 | C9 | 0.014 |
| chr5:39316180 | C9 | 0.0007194 |
| chr5:39316274 | C9 | 0.016 |
| chr5:39316422 | C9 | 0.333 |
| chr5:39331765 | C9 | 0.0007184 |
| chr5:39331786 | C9 | 0.0007184 |
| chr5:39331859 | C9 | 0.007194 |
| chr5:39331894 | C9 | 0.0007194 |
| chr5:39332103 | C9 | 0.0007194 |
| chr5:39341009 | C9 | 0.087 |
| chr5:39341083 | C9 | 0.0007194 |
| chr5:39341337 | C9 | 0.019 |
| chr5:39341518 | C9 | 0.001437 |
| chr5:39341520 | C9 | 0.007902 |
| chr5:39341523 | C9 | 0.002155 |
| chr5:39341801 | C9 | 0.0007184 |
| chr5:39342049 | C9 | 0.003602 |
| chr5:39342119 | C9 | 0.0007194 |
| chr5:39342214 | C9 | 0.001439 |
| chr5:39342243 | C9 | 0.002158 |
| chr5:39342308 | C9 | 0.402 |
| chr5:39342493 | C9 | 0.773 |
| chr5:39364339 | C9 | 0.377 |
| chr5:39364358 | C9 | 0.381 |
| chr5:39364474 | C9 | 0.085 |
| chr5:39364554 | C9 | 0.371 |
| chr5:39364643 | C9 | 0.0007184 |
| chr5:39364748 | C9 | 0.0007194 |
| chr5:40909771 | C7 | 0.0007194 |
| chr5:40928486 | C7 | 0.012 |
| chr5:40928574 | C7 | 0.41 |
| chr5:40928766 | C7 | 0.0007519 |
| chr5:40928792 | C7 | 0.377 |
| chr5:40928803 | C7 | 0.0007813 |
| chr5:40931062 | C7 | 0.002155 |
| chr5:40931115 | C7 | 0.0007184 |

| | | |
|---|---|---|
| chr5:40931357 | C7 | 0.197 |
| chr5:40934154 | C7 | 0.046 |
| chr5:40934166 | C7 | 0.096 |
| chr5:40934201 | C7 | 0.0007911 |
| chr5:40934471 | C7 | 0.005747 |
| chr5:40934549 | C7 | 0.0007194 |
| chr5:40934767 | C7 | 0.004367 |
| chr5:40934832 | C7 | 0.137 |
| chr5:40936297 | C7 | 0.001439 |
| chr5:40936541 | C7 | 0.01 |
| chr5:40936669 | C7 | 0.129 |
| chr5:40936791 | C7 | 0.132 |
| chr5:40936843 | C7 | 0.397 |
| chr5:40936861 | C7 | 0.081 |
| chr5:40937464 | C7 | 0.005051 |
| chr5:40937657 | C7 | 0.005764 |
| chr5:40937768 | C7 | 0.0007194 |
| chr5:40937803 | C7 | 0.0007194 |
| chr5:40938000 | C7 | 0.205 |
| chr5:40945337 | C7 | 0.0007215 |
| chr5:40945432 | C7 | 0.0007194 |
| chr5:40947535 | C7 | 0.002882 |
| chr5:40947571 | C7 | 0.001441 |
| chr5:40948139 | C7 | 0.582 |
| chr5:40949776 | C7 | 0.664 |
| chr5:40949804 | C7 | 0.13 |
| chr5:40949997 | C7 | 0.662 |
| chr5:40950059 | C7 | 0.0007194 |
| chr5:40950187 | C7 | 0.0007225 |
| chr5:40950248 | C7 | 0.0007225 |
| chr5:40955281 | C7 | 0.0007205 |
| chr5:40955367 | C7 | 0.0007194 |
| chr5:40955561 | C7 | 0.629 |
| chr5:40955711 | C7 | 0.0007184 |
| chr5:40955940 | C7 | 0.002551 |
| chr5:40957874 | C7 | 0.728 |
| chr5:40958027 | C7 | 1 |
| chr5:40958031 | C7 | 0.029 |
| chr5:40958502 | C7 | 0.0007184 |
| chr5:40959407 | C7 | 0.0007194 |
| chr5:40959622 | C7 | 0.001435 |
| chr5:40959735 | C7 | 0.002874 |
| chr5:40959959 | C7 | 0.166 |
| chr5:40961894 | C7 | 0.77 |
| chr5:40961989 | C7 | 0.0007257 |
| chr5:40962083 | C7 | 0.014 |
| chr5:40962547 | C7 | 0.772 |
| chr5:40964557 | C7 | 0.125 |
| chr5:40964595 | C7 | 0.223 |
| chr5:40964682 | C7 | 0.763 |
| chr5:40964684 | C7 | 0.764 |

| | | |
|---|---|---|
| chr5:40964784 | C7 | 0.0007205 |
| chr5:40965103 | C7 | 0.0007194 |
| chr5:40972211 | C7 | 0.094 |
| chr5:40972371 | C7 | 0.257 |
| chr5:40972534 | C7 | 0.0007194 |
| chr5:40972630 | C7 | 0.0007194 |
| chr5:40976669 | C7 | 0.772 |
| chr5:40976773 | C7 | 0.0007194 |
| chr5:40976824 | C7 | 0.0007194 |
| chr5:40977136 | C7 | 0.002882 |
| chr5:40979593 | C7 | 0.079 |
| chr5:40979604 | C7 | 0.249 |
| chr5:40979662 | C7 | 0.43 |
| chr5:40979749 | C7 | 0.0007194 |
| chr5:40979790 | C7 | 0.249 |
| chr5:40979901 | C7 | 0.0007184 |
| chr5:40980086 | C7 | 0.428 |
| chr5:40980102 | C7 | 0.248 |
| chr5:40980166 | C7 | 0.099 |
| chr5:40980231 | C7 | 0.0007205 |
| chr5:40981391 | C7 | 0.0007194 |
| chr5:40981399 | C7 | 0.002878 |
| chr5:40981689 | C7 | 0.43 |
| chr5:40981701 | C7 | 0.00431 |
| chr5:40981768..40981771 | C7 | 0.245 |
| chr5:40981927 | C7 | 0.011 |
| chr5:41142932 | C6 | 0.0007194 |
| chr5:41149080 | C6 | 0.027 |
| chr5:41149147 | C6 | 0.233 |
| chr5:41149235 | C6 | 0.234 |
| chr5:41149284 | C6 | 0.159 |
| chr5:41149361 | C6 | 0.001439 |
| chr5:41149448 | C6 | 0.014 |
| chr5:41149768 | C6 | 0.02 |
| chr5:41149952 | C6 | 0.0007184 |
| chr5:41150035 | C6 | 0.0007194 |
| chr5:41150426 | C6 | 0.051 |
| chr5:41154150 | C6 | 0.827 |
| chr5:41154241 | C6 | 0.002158 |
| chr5:41154384 | C6 | 0.017 |
| chr5:41154879 | C6 | 0.019 |
| chr5:41155088 | C6 | 0.007902 |
| chr5:41155447 | C6 | 0.001486 |
| chr5:41158489 | C6 | 0.146 |
| chr5:41158572 | C6 | 0.828 |
| chr5:41158671 | C6 | 0.822 |
| chr5:41158863 | C6 | 1 |
| chr5:41158922 | C6 | 0.0007205 |
| chr5:41158956 | C6 | 0.0007215 |
| chr5:41158967 | C6 | 0.01 |
| chr5:41158981 | C6 | 0.026 |

| | | |
|---|---|---|
| chr5:41158986 | C6 | 0.022 |
| chr5:41159496 | C6 | 0.012 |
| chr5:41159509 | C6 | 0.145 |
| chr5:41159529 | C6 | 0.008633 |
| chr5:41159653 | C6 | 0.149 |
| chr5:41160065 | C6 | 0.146 |
| chr5:41160293 | C6 | 0.029 |
| chr5:41161521 | C6 | 0.825 |
| chr5:41161648 | C6 | 0.001441 |
| chr5:41161898 | C6 | 0.009353 |
| chr5:41162198 | C6 | 0.001484 |
| chr5:41172080 | C6 | 0.001486 |
| chr5:41176454 | C6 | 0.001453 |
| chr5:41177016..41177017 | C6 | 0.45 |
| chr5:41181578 | C6 | 0.007184 |
| chr5:41181636 | C6 | 0.0007184 |
| chr5:41185912 | C6 | 0.079 |
| chr5:41186186 | C6 | 0.002155 |
| chr5:41186220 | C6 | 0.032 |
| chr5:41186248 | C6 | 0.001437 |
| chr5:41186449 | C6 | 0.48 |
| chr5:41186505 | C6 | 0.0007236 |
| chr5:41195700 | C6 | 0.079 |
| chr5:41195783 | C6 | 0.003597 |
| chr5:41195860 | C6 | 0.0007184 |
| chr5:41195939 | C6 | 0.012 |
| chr5:41195986 | C6 | 0.006475 |
| chr5:41196079 | C6 | 0.017 |
| chr5:41196190 | C6 | 0.013 |
| chr5:41196230 | C6 | 0.078 |
| chr5:41196273 | C6 | 0.037 |
| chr5:41196311 | C6 | 0.149 |
| chr5:41199616 | C6 | 0.662 |
| chr5:41199629 | C6 | 0.669 |
| chr5:41199631 | C6 | 0.0007849 |
| chr5:41199752 | C6 | 0.024 |
| chr5:41199959 | C6 | 0.613 |
| chr5:41201426 | C6 | 0.033 |
| chr5:41201488 | C6 | 0.0007194 |
| chr5:41201759..41201760 | C6 | 0.0007184 |
| chr5:41201807 | C6 | 0.0007194 |
| chr5:41203034 | C6 | 0.0007194 |
| chr5:41203164 | C6 | 0.002158 |
| chr5:41203322 | C6 | 0.0007184 |
| chr5:41203344 | C6 | 0.078 |
| chr5:41203547 | C6 | 0.035 |
| chr6:31895422 | C2 | 0.0007194 |
| chr6:31895494 | C2 | 0.001437 |
| chr6:31895591 | C2 | 0.007902 |
| chr6:31895619 | C2 | 0.0007184 |
| chr6:31895758 | C2 | 0.005029 |

| | | |
|---|---|---|
| chr6:31895973 | C2 | 0.231 |
| chr6:31896112 | C2 | 0.0007194 |
| chr6:31896279 | C2 | 0.001443 |
| chr6:31896368..31896370 | C2 | 0.0007194 |
| chr6:31896478 | C2 | 0.0007184 |
| chr6:31896873 | C2 | 0.00938 |
| chr6:31896874 | C2 | 0.0007215 |
| chr6:31901090 | C2 | 0.035 |
| chr6:31901264 | C2 | 0.011 |
| chr6:31901536 | C2 | 0.0007194 |
| chr6:31901895 | C2 | 0.006456 |
| chr6:31902289 | C2 | 0.04 |
| chr6:31902377 | C2 | 0.0007205 |
| chr6:31903931 | C2 | 0.018 |
| chr6:31903959 | C2 | 0.0007184 |
| chr6:31904802 | C2 | 0.002433 |
| chr6:31905130 | C2 | 0.068 |
| chr6:31905293 | C2 | 0.01 |
| chr6:31906828 | C2 | 0.037 |
| chr6:31906865 | C2 | 0.069 |
| chr6:31906905 | C2 | 0.0007194 |
| chr6:31907168 | C2 | 0.068 |
| chr6:31910456 | C2 | 0.005029 |
| chr6:31910557 | C2 | 0.0007184 |
| chr6:31910929 | C2 | 0.069 |
| chr6:31910938 | C2 | 0.069 |
| chr6:31911583 | C2 | 0.0007184 |
| chr6:31911809 | C2 | 0.006475 |
| chr6:31912009 | C2 | 0.143 |
| chr6:31912375 | C2 | 0.0007194 |
| chr6:31912392 | C2 | 0.033 |
| chr6:31912478 | C2 | 0.0007194 |
| chr6:31912523 | C2 | 0.002874 |
| chr6:31912773 | C2 | 0.007902 |
| chr6:31912858 | C2 | 0.001437 |
| chr6:31912947 | C2 | 0.0007184 |
| chr6:31913216 | C2 | 0.0007174 |
| chr6:31914024 | CFB | 0.052 |
| chr6:31914305 | CFB | 0.0007184 |
| chr6:31914669 | CFB | 0.007902 |
| chr6:31914673 | CFB | 0.007184 |
| chr6:31914791 | CFB | 0.0007184 |
| chr6:31914847 | CFB | 0.0007184 |
| chr6:31914935 | CFB | 0.826 |
| chr6:31915003 | CFB | 0.005747 |
| chr6:31915102 | CFB | 0.0007184 |
| chr6:31915144 | CFB | 0.052 |
| chr6:31915240 | CFB | 0.005755 |
| chr6:31915387 | CFB | 0.0007184 |
| chr6:31915532 | CFB | 0.034 |
| chr6:31915584 | CFB | 0.0007184 |

| | | |
|---|---|---|
| chr6:31915614 | CFB | 0.021 |
| chr6:31916013 | CFB | 0.035 |
| chr6:31916062 | CFB | 0.003592 |
| chr6:31916400 | CFB | 0.699 |
| chr6:31916491..31916494 | CFB | 0.032 |
| chr6:31916713 | CFB | 0.001439 |
| chr6:31916912 | CFB | 0.0007184 |
| chr6:31916916 | CFB | 0.0007184 |
| chr6:31916951 | CFB | 0.07 |
| chr6:31916985 | CFB | 0.002874 |
| chr6:31917002 | CFB | 0.002158 |
| chr6:31917048 | CFB | 0.0007194 |
| chr6:31917291 | CFB | 0.032 |
| chr6:31917405 | CFB | 0.0007184 |
| chr6:31917540 | CFB | 0.362 |
| chr6:31917557 | CFB | 0.07 |
| chr6:31917709 | CFB | 0.001437 |
| chr6:31918080 | CFB | 0.002155 |
| chr6:31918209 | CFB | 0.0007184 |
| chr6:31918224 | CFB | 0.0007184 |
| chr6:31918252 | CFB | 0.0007184 |
| chr6:31918464 | CFB | 0.004317 |
| chr6:31918468 | CFB | 0.0007194 |
| chr6:31918558 | CFB | 0.013 |
| chr6:31918737 | CFB | 0.0007194 |
| chr6:31918860 | CFB | 0.092 |
| chr6:31918903 | CFB | 0.052 |
| chr6:31919578 | CFB | 0.627 |
| chr6:31919830 | CFB | 0.052 |
| chr6:31919854 | CFB | 0.002155 |
| chr6:31919855 | CFB | 0.0007184 |
| chr6:31961864 | C4A | 0.02 |
| chr6:31963820 | C4A | 0.0007246 |
| chr7:100771717 | SERPINE1 | 0.077 |
| chr7:100771723 | SERPINE1 | 0.039 |
| chr7:100772139 | SERPINE1 | 0.006475 |
| chr7:100772145 | SERPINE1 | 0.0007194 |
| chr7:100773451 | SERPINE1 | 0.012 |
| chr7:100773459 | SERPINE1 | 0.842 |
| chr7:100773883 | SERPINE1 | 0.007194 |
| chr7:100774008 | SERPINE1 | 0.009353 |
| chr7:100774870 | SERPINE1 | 0.999 |
| chr7:100775018 | SERPINE1 | 0.024 |
| chr7:100775259 | SERPINE1 | 0.0007184 |
| chr7:100775352 | SERPINE1 | 0.0007174 |
| chr7:100775449 | SERPINE1 | 0.0007184 |
| chr7:100776953 | SERPINE1 | 0.0007184 |
| chr7:100777197 | SERPINE1 | 0.002874 |
| chr7:100777372 | SERPINE1 | 0.01 |
| chr7:100779015 | SERPINE1 | 0.005029 |
| chr7:100779244 | SERPINE1 | 0.106 |

| | | |
|---|---|---|
| chr7:100779297 | SERPINE1 | 0.0007205 |
| chr7:100780233 | SERPINE1 | 0.003597 |
| chr7:100780385 | SERPINE1 | 0.0007184 |
| chr7:100780903 | SERPINE1 | 0.002158 |
| chr7:100780956 | SERPINE1 | 0.0007236 |
| chr8:27455919 | CLU | 0.001437 |
| chr8:27456253 | CLU | 0.613 |
| chr8:27456433..27456435 | CLU | 0.194 |
| chr8:27457193 | CLU | 0.0007184 |
| chr8:27457246 | CLU | 0.0007194 |
| chr8:27457282 | CLU | 0.0007184 |
| chr8:27457414 | CLU | 0.0007174 |
| chr8:27457477 | CLU | 0.004317 |
| chr8:27457512 | CLU | 0.0007194 |
| chr8:27457577 | CLU | 0.0007184 |
| chr8:27457583 | CLU | 0.0007184 |
| chr8:27457673 | CLU | 0.0007184 |
| chr8:27457748 | CLU | 0.007914 |
| chr8:27457793 | CLU | 0.001502 |
| chr8:27461590 | CLU | 0.001439 |
| chr8:27461772..27461773 | CLU | 0.335 |
| chr8:27461853 | CLU | 0.001437 |
| chr8:27462008 | CLU | 0.196 |
| chr8:27462150 | CLU | 0.23 |
| chr8:27462290 | CLU | 0.002874 |
| chr8:27462461 | CLU | 0.0007184 |
| chr8:27462481 | CLU | 0.616 |
| chr8:27462662 | CLU | 0.001435 |
| chr8:27462725 | CLU | 0.003592 |
| chr8:27463137 | CLU | 0.01 |
| chr8:27463150 | CLU | 0.002451 |
| chr8:27463618 | CLU | 0.418 |
| chr8:27463639 | CLU | 0.0007205 |
| chr8:27463746 | CLU | 0.0007194 |
| chr8:27464076 | CLU | 0.007891 |
| chr8:27464207 | CLU | 0.017 |
| chr8:27464244 | CLU | 0.199 |
| chr8:27466157 | CLU | 0.61 |
| chr8:27466181 | CLU | 0.607 |
| chr8:27466315 | CLU | 0.615 |
| chr8:27466836 | CLU | 0.0007205 |
| chr8:27467705 | CLU | 0.00578 |
| chr8:27467821 | CLU | 0.608 |
| chr8:27467843 | CLU | 0.002155 |
| chr8:27468005 | CLU | 0.0007194 |
| chr8:27468862 | CLU | 0.341 |
| chr8:27469168 | CLU | 0.003597 |
| chr8:27469350 | CLU | 0.0007205 |
| chr8:27471924 | CLU | 0.012 |
| chr8:27472251 | CLU | 0.001437 |
| chr9:123715167 | C5 | 0.002155 |

| | | |
|---|---|---|
| chr9:123715210 | C5 | 0.017 |
| chr9:123715906 | C5 | 0.0007194 |
| chr9:123715951 | C5 | 0.0007184 |
| chr9:123716103 | C5 | 0.014 |
| chr9:123716229 | C5 | 0.0007194 |
| chr9:123719334 | C5 | 0.002294 |
| chr9:123719335 | C5 | 0.038 |
| chr9:123719681 | C5 | 0.0007194 |
| chr9:123719835 | C5 | 0.001445 |
| chr9:123722359 | C5 | 0.043 |
| chr9:123723534 | C5 | 0.014 |
| chr9:123724926 | C5 | 0.001439 |
| chr9:123725524 | C5 | 0.01 |
| chr9:123725677 | C5 | 0.018 |
| chr9:123725776 | C5 | 0.014 |
| chr9:123725808 | C5 | 0.00219 |
| chr9:123725926 | C5 | 0.014 |
| chr9:123725971 | C5 | 0.027 |
| chr9:123726029 | C5 | 0.014 |
| chr9:123726145 | C5 | 0.028 |
| chr9:123731217 | C5 | 0.002894 |
| chr9:123731408 | C5 | 0.014 |
| chr9:123732662 | C5 | 0.002158 |
| chr9:123734287..123734291 | C5 | 0.109 |
| chr9:123734518 | C5 | 0.022 |
| chr9:123737018 | C5 | 0.11 |
| chr9:123737040 | C5 | 0.157 |
| chr9:123737203 | C5 | 0.001439 |
| chr9:123739337 | C5 | 0.002161 |
| chr9:123742482 | C5 | 0.001437 |
| chr9:123742818 | C5 | 0.603 |
| chr9:123743921 | C5 | 0.045 |
| chr9:123743922 | C5 | 0.0007205 |
| chr9:123744252 | C5 | 0.045 |
| chr9:123744275 | C5 | 0.0007194 |
| chr9:123744497 | C5 | 0.049 |
| chr9:123751433 | C5 | 0.0007184 |
| chr9:123751648 | C5 | 0.052 |
| chr9:123751661 | C5 | 0.049 |
| chr9:123751728..123751729 | C5 | 0.002525 |
| chr9:123751800 | C5 | 0.592 |
| chr9:123753253 | C5 | 0.05 |
| chr9:123753600 | C5 | 0.052 |
| chr9:123758632 | C5 | 0.05 |
| chr9:123759608 | C5 | 0.016 |
| chr9:123759929 | C5 | 0.0007184 |
| chr9:123760107 | C5 | 0.504 |
| chr9:123760323 | C5 | 0.529 |
| chr9:123761900..123761902 | C5 | 0.027 |
| chr9:123762321 | C5 | 0.494 |
| chr9:123762487 | C5 | 0.0007215 |

| | | |
|---|---|---|
| chr9:123769200 | C5 | 0.489 |
| chr9:123769460 | C5 | 0.0007692 |
| chr9:123770436 | C5 | 0.617 |
| chr9:123770793 | C5 | 0.0007194 |
| chr9:123771010 | C5 | 0.073 |
| chr9:123775851 | C5 | 0.001779 |
| chr9:123776007 | C5 | 0.061 |
| chr9:123776047 | C5 | 0.024 |
| chr9:123776132 | C5 | 0.0007174 |
| chr9:123776369 | C5 | 0.0007194 |
| chr9:123776390 | C5 | 0.059 |
| chr9:123776572 | C5 | 0.0007205 |
| chr9:123777733 | C5 | 0.001462 |
| chr9:123778688 | C5 | 0.059 |
| chr9:123778787 | C5 | 0.0007205 |
| chr9:123779553 | C5 | 0.002158 |
| chr9:123779705 | C5 | 0.0007194 |
| chr9:123780005 | C5 | 0.488 |
| chr9:123782299 | C5 | 0.06 |
| chr9:123783899 | C5 | 0.0007194 |
| chr9:123783934 | C5 | 0.124 |
| chr9:123784015 | C5 | 0.0007194 |
| chr9:123785535 | C5 | 0.0007194 |
| chr9:123785545 | C5 | 0.124 |
| chr9:123785658 | C5 | 0.001439 |
| chr9:123785738 | C5 | 0.027 |
| chr9:123786035 | C5 | 0.13 |
| chr9:123787566 | C5 | 0.603 |
| chr9:123787580..123787582 | C5 | 0.005556 |
| chr9:123787743 | C5 | 0.001441 |
| chr9:123787999 | C5 | 0.086 |
| chr9:123789188 | C5 | 0.0007194 |
| chr9:123789634 | C5 | 0.485 |
| chr9:123792469 | C5 | 0.061 |
| chr9:123792906 | C5 | 0.124 |
| chr9:123792989 | C5 | 0.059 |
| chr9:123794383 | C5 | 0.001441 |
| chr9:123794682 | C5 | 0.008929 |
| chr9:123794756 | C5 | 0.083 |
| chr9:123797324 | C5 | 0.714 |
| chr9:123797428 | C5 | 0.733 |
| chr9:123800094 | C5 | 0.488 |
| chr9:123800105 | C5 | 0.021 |
| chr9:123800211 | C5 | 0.0007576 |
| chr9:123800218 | C5 | 0.059 |
| chr9:123800426 | C5 | 0.015 |
| chr9:123805440 | C5 | 0.0007215 |
| chr9:123808220 | C5 | 0.782 |
| chr9:123808249 | C5 | 0.053 |
| chr9:123808262 | C5 | 0.137 |
| chr9:123812236..123812237 | C5 | 0.695 |

| | | |
|---|---|---|
| chr9:123812499 | C5 | 0.0007194 |
| chr9:123812548 | C5 | 0.0007194 |
| chr9:123812823 | C5 | 0.003125 |
| chr9:139839832 | C8G | 0.001437 |
| chr9:139839904 | C8G | 0.5 |
| chr9:139839922 | C8G | 0.0007184 |
| chr9:139839974..139839977 | C8G | 0.101 |
| chr9:139840258 | C8G | 0.001439 |
| chr9:139840333 | C8G | 0.056 |
| chr9:139840378 | C8G | 0.041 |
| chr9:139840504 | C8G | 0.0007194 |
| chr9:139840543 | C8G | 0.949 |
| chr9:139840560 | C8G | 0.1 |
| chr9:139840580 | C8G | 0.1 |
| chr9:139840680 | C8G | 0.0007194 |
| chr9:139840817 | C8G | 0.0007174 |
| chr9:139840859 | C8G | 0.949 |
| chr9:139841132 | C8G | 0.001437 |
| chr9:139841212 | C8G | 0.1 |
| chr9:139841369 | C8G | 0.001439 |
| chr10:54527921 | MBL2 | 0.022 |
| chr10:54528161 | MBL2 | 0.0007184 |
| chr10:54528236 | MBL2 | 0.065 |
| chr10:54528353 | MBL2 | 0.786 |
| chr10:54528746 | MBL2 | 0.006686 |
| chr10:54528835 | MBL2 | 0.001441 |
| chr10:54529257 | MBL2 | 0.427 |
| chr10:54529259 | MBL2 | 0.005755 |
| chr10:54529325 | MBL2 | 0.445 |
| chr10:54530260 | MBL2 | 0.001439 |
| chr10:54530621 | MBL2 | 0.0007194 |
| chr10:54530642 | MBL2 | 0.032 |
| chr10:54530664 | MBL2 | 0.0007194 |
| chr10:54530694 | MBL2 | 1 |
| chr10:54530843 | MBL2 | 0.176 |
| chr10:54531084 | MBL2 | 0.011 |
| chr10:54531226 | MBL2 | 0.007184 |
| chr10:54531235 | MBL2 | 0.126 |
| chr10:54531242 | MBL2 | 0.055 |
| chr10:54531352 | MBL2 | 0.0007184 |
| chr10:54531461 | MBL2 | 0.175 |
| chr10:54531490 | MBL2 | 0.007194 |
| chr10:54531534 | MBL2 | 0.177 |
| chr10:54531685 | MBL2 | 0.786 |
| chr11:33731406 | CD59 | 0.302 |
| chr11:33731545 | CD59 | 0.019 |
| chr11:33731624 | CD59 | 0.007914 |
| chr11:33733196 | CD59 | 0.024 |
| chr11:33738764 | CD59 | 0.0007205 |
| chr11:33739126 | CD59 | 0.0007194 |
| chr11:33743679 | CD59 | 0.378 |

| | | |
|---|---|---|
| chr11:33743702 | CD59 | 0.0007184 |
| chr11:33743974 | CD59 | 0.0007184 |
| chr11:33744153 | CD59 | 0.001439 |
| chr11:33744254 | CD59 | 0.03 |
| chr11:57365723 | SERPING1 | 0.06 |
| chr11:57365748 | SERPING1 | 0.002874 |
| chr11:57365895 | SERPING1 | 0.14 |
| chr11:57366092 | SERPING1 | 0.02 |
| chr11:57367222 | SERPING1 | 0.32 |
| chr11:57367312 | SERPING1 | 0.0007194 |
| chr11:57367442 | SERPING1 | 0.001439 |
| chr11:57369328 | SERPING1 | 0.273 |
| chr11:57369355 | SERPING1 | 0.0007194 |
| chr11:57369466 | SERPING1 | 0.002158 |
| chr11:57369730 | SERPING1 | 0.276 |
| chr11:57373304 | SERPING1 | 0.0007194 |
| chr11:57373548 | SERPING1 | 0.01 |
| chr11:57373646 | SERPING1 | 0.001437 |
| chr11:57374074 | SERPING1 | 0.0007184 |
| chr11:57374080 | SERPING1 | 0.0007184 |
| chr11:57374104 | SERPING1 | 0.001437 |
| chr11:57379170 | SERPING1 | 0.691 |
| chr11:57381519 | SERPING1 | 0.401 |
| chr11:57381989 | SERPING1 | 0.293 |
| chr12:7169362 | C1S | 0.028 |
| chr12:7169473 | C1S | 0.013 |
| chr12:7169661 | C1S | 0.178 |
| chr12:7169873 | C1S | 0.0007194 |
| chr12:7170118 | C1S | 0.0007194 |
| chr12:7170145 | C1S | 0.002158 |
| chr12:7170171 | C1S | 0.041 |
| chr12:7170336 | C1S | 0.176 |
| chr12:7171282 | C1S | 0.0007289 |
| chr12:7171454 | C1S | 0.0007184 |
| chr12:7171507 | C1S | 0.224 |
| chr12:7171620 | C1S | 0.075 |
| chr12:7172241 | C1S | 0.256 |
| chr12:7172902 | C1S | 0.185 |
| chr12:7173413 | C1S | 0.001439 |
| chr12:7173893 | C1S | 0.002155 |
| chr12:7174770 | C1S | 0.952 |
| chr12:7175047 | C1S | 0.075 |
| chr12:7175117 | C1S | 0.0007184 |
| chr12:7175298 | C1S | 0.002874 |
| chr12:7175628 | C1S | 0.002158 |
| chr12:7175782 | C1S | 0.003592 |
| chr12:7175872 | C1S | 0.179 |
| chr12:7175979 | C1S | 0.009353 |
| chr12:7176978 | C1S | 0.178 |
| chr12:7176984 | C1S | 0.18 |
| chr12:7177171 | C1S | 0.003597 |

| | | |
|---|---|---|
| chr12:7177565 | C1S | 0.00431 |
| chr12:7177779 | C1S | 0.001439 |
| chr12:7178019 | C1S | 0.176 |
| chr12:7178060 | C1S | 0.0007184 |
| chr12:7187646 | C1R | 0.0007184 |
| chr12:7188562 | C1R | 0.015 |
| chr12:7189687 | C1R | 0.001595 |
| chr12:7240932 | C1R | 0.001563 |
| chr12:7241055 | C1R | 0.084 |
| chr12:7241086 | C1R | 0.01 |
| chr12:7241090 | C1R | 0.005755 |
| chr12:7241570 | C1R | 0.01 |
| chr12:7241751 | C1R | 0.0007194 |
| chr12:7242204 | C1R | 0.068 |
| chr12:7242299 | C1R | 0.307 |
| chr12:7242414 | C1R | 0.0007184 |
| chr12:7242740 | C1R | 0.00431 |
| chr12:7242821 | C1R | 0.002158 |
| chr12:7242869..7242872 | C1R | 0.0007184 |
| chr12:7243783 | C1R | 0.282 |
| chr12:7243895 | C1R | 0.0007205 |
| chr12:7243916 | C1R | 0.0007194 |
| chr12:7243933 | C1R | 0.0007194 |
| chr12:7244286 | C1R | 0.0007194 |
| chr12:7244382 | C1R | 0.024 |
| chr12:7244549 | C1R | 0.001439 |
| chr12:7244611 | C1R | 0.0007194 |
| chr12:7245030 | C1R | 0.0007184 |
| chr12:7245097 | C1R | 0.0007194 |
| chr12:8211117 | C3AR1 | 0.004329 |
| chr12:8211187 | C3AR1 | 0.278 |
| chr12:8211436 | C3AR1 | 0.0007184 |
| chr12:8211779 | C3AR1 | 0.0007184 |
| chr12:8211784 | C3AR1 | 0.026 |
| chr12:8212512 | C3AR1 | 0.002155 |
| chr14:94844562 | SERPINA1 | 0.255 |
| chr14:94844706 | SERPINA1 | 0.078 |
| chr14:94844843 | SERPINA1 | 0.256 |
| chr14:94844884 | SERPINA1 | 0.0007184 |
| chr14:94844947 | SERPINA1 | 0.019 |
| chr14:94845077 | SERPINA1 | 0.0007194 |
| chr14:94845193 | SERPINA1 | 0.0007194 |
| chr14:94845277 | SERPINA1 | 0.007959 |
| chr14:94845592 | SERPINA1 | 0.003597 |
| chr14:94845834 | SERPINA1 | 0.0007194 |
| chr14:94845944 | SERPINA1 | 0.001437 |
| chr14:94846943 | SERPINA1 | 0.282 |
| chr14:94846981 | SERPINA1 | 0.0007205 |
| chr14:94847012 | SERPINA1 | 0.0007184 |
| chr14:94847262 | SERPINA1 | 0.01 |
| chr14:94847305 | SERPINA1 | 0.0007194 |

| | | |
|---|---|---|
| chr14:94847415 | SERPINA1 | 0.282 |
| chr14:94847500 | SERPINA1 | 0.0007194 |
| chr14:94847549 | SERPINA1 | 0.0007194 |
| chr14:94847720 | SERPINA1 | 0.002161 |
| chr14:94848738 | SERPINA1 | 0.002882 |
| chr14:94848849 | SERPINA1 | 0.003597 |
| chr14:94849151 | SERPINA1 | 0.021 |
| chr14:94849201 | SERPINA1 | 0.116 |
| chr14:94849325 | SERPINA1 | 0.0007194 |
| chr14:94849532 | SERPINA1 | 0.002158 |
| chr14:94849769 | SERPINA1 | 0.0007184 |
| chr14:95053890 | SERPINA5 | 0.633 |
| chr14:95054012 | SERPINA5 | 0.111 |
| chr14:95054087 | SERPINA5 | 0.001437 |
| chr14:95054472 | SERPINA5 | 0.001439 |
| chr14:95054521 | SERPINA5 | 0.0007194 |
| chr14:95054538 | SERPINA5 | 0.002161 |
| chr14:95054577 | SERPINA5 | 0.001462 |
| chr14:95056113 | SERPINA5 | 0.482 |
| chr14:95056213 | SERPINA5 | 0.001439 |
| chr14:95056278 | SERPINA5 | 0.0007194 |
| chr14:95056440 | SERPINA5 | 0.002155 |
| chr14:95056501 | SERPINA5 | 0.0007184 |
| chr14:95056628 | SERPINA5 | 0.0007184 |
| chr14:95057294 | SERPINA5 | 0.078 |
| chr14:95057498..95057501 | SERPINA5 | 0.052 |
| chr14:95057512..95057515 | SERPINA5 | 0.066 |
| chr14:95058114 | SERPINA5 | 0.003644 |
| chr14:95058360 | SERPINA5 | 0.684 |
| chr14:95058390 | SERPINA5 | 0.0007184 |
| chr14:95058462 | SERPINA5 | 0.486 |
| chr14:95058525 | SERPINA5 | 0.0007194 |
| chr14:95058614 | SERPINA5 | 0.024 |
| chr14:95058631 | SERPINA5 | 0.555 |
| chr14:95058642 | SERPINA5 | 0.012 |
| chr14:95058737 | SERPINA5 | 0.051 |
| chr14:95058813 | SERPINA5 | 0.48 |
| chr17:5336188 | C1QBP | 0.319 |
| chr17:5336210 | C1QBP | 0.318 |
| chr17:5336215 | C1QBP | 0.044 |
| chr17:5336492 | C1QBP | 0.007914 |
| chr17:5336791 | C1QBP | 0.314 |
| chr17:5338408 | C1QBP | 0.0007194 |
| chr17:5338460 | C1QBP | 0.002878 |
| chr17:5341429 | C1QBP | 0.0007184 |
| chr17:5341683 | C1QBP | 0.004323 |
| chr17:5341749 | C1QBP | 0.441 |
| chr17:5341874 | C1QBP | 0.122 |
| chr17:26694483 | VTN | 0.096 |
| chr17:26694529 | VTN | 0.0007194 |
| chr17:26694660 | VTN | 0.0007194 |

| | | |
|---|---|---|
| chr17:26694661 | VTN | 0.096 |
| chr17:26695467 | VTN | 0.027 |
| chr17:26695704 | VTN | 0.946 |
| chr17:26695759 | VTN | 0.0007184 |
| chr17:26695832 | VTN | 0.552 |
| chr17:26696156 | VTN | 0.0007194 |
| chr17:26696477 | VTN | 0.107 |
| chr17:26696482 | VTN | 0.107 |
| chr17:26696887 | VTN | 0.0007194 |
| chr17:26696918 | VTN | 0.001439 |
| chr17:26697451 | VTN | 0.114 |
| chr17:26697521 | VTN | 0.096 |
| chr17:26694861 | CTB-96E2.2 | 0.001439 |
| chr18:61558502 | SERPINB2 | 0.0007194 |
| chr18:61558612 | SERPINB2 | 0.002158 |
| chr18:61558613 | SERPINB2 | 0.221 |
| chr18:61558656 | SERPINB2 | 0.219 |
| chr18:61558878 | SERPINB2 | 0.219 |
| chr18:61558882 | SERPINB2 | 0.022 |
| chr18:61559028 | SERPINB2 | 0.257 |
| chr18:61559135 | SERPINB2 | 0.001439 |
| chr18:61562348 | SERPINB2 | 0.22 |
| chr18:61562391 | SERPINB2 | 0.0007184 |
| chr18:61562424 | SERPINB2 | 0.001437 |
| chr18:61562568 | SERPINB2 | 0.002874 |
| chr18:61562580 | SERPINB2 | 0.0007184 |
| chr18:61562608 | SERPINB2 | 0.221 |
| chr18:61562690 | SERPINB2 | 0.219 |
| chr18:61562789 | SERPINB2 | 0.219 |
| chr18:61562797 | SERPINB2 | 0.231 |
| chr18:61564106 | SERPINB2 | 0.227 |
| chr18:61564114 | SERPINB2 | 0.004399 |
| chr18:61564208 | SERPINB2 | 0.219 |
| chr18:61564394 | SERPINB2 | 0.001439 |
| chr18:61564441 | SERPINB2 | 0.218 |
| chr18:61564532 | SERPINB2 | 0.22 |
| chr18:61564589 | SERPINB2 | 0.004317 |
| chr18:61564599 | SERPINB2 | 0.219 |
| chr18:61564610 | SERPINB2 | 0.0007205 |
| chr18:61564696 | SERPINB2 | 0.22 |
| chr18:61564779 | SERPINB2 | 0.016 |
| chr18:61564786 | SERPINB2 | 0.0007194 |
| chr18:61565014 | SERPINB2 | 0.027 |
| chr18:61565062 | SERPINB2 | 0.222 |
| chr18:61565208 | SERPINB2 | 0.226 |
| chr18:61568869 | SERPINB2 | 0.014 |
| chr18:61569091 | SERPINB2 | 0.032 |
| chr18:61569364 | SERPINB2 | 0.233 |
| chr18:61569383 | SERPINB2 | 0.026 |
| chr18:61569463 | SERPINB2 | 0.225 |
| chr18:61569543 | SERPINB2 | 0.0007205 |

| | | |
|---|---|---|
| chr18:61569559 | SERPINB2 | 0.016 |
| chr18:61569598 | SERPINB2 | 0.002158 |
| chr18:61569645 | SERPINB2 | 0.0007184 |
| chr18:61569689 | SERPINB2 | 0.0007184 |
| chr18:61569697 | SERPINB2 | 0.22 |
| chr18:61569819..61569834 | SERPINB2 | 0.22 |
| chr18:61569849..61569854 | SERPINB2 | 0.0007205 |
| chr18:61569980 | SERPINB2 | 0.0007205 |
| chr18:61570004 | SERPINB2 | 0.0007184 |
| chr18:61570300 | SERPINB2 | 0.191 |
| chr18:61570470 | SERPINB2 | 0.219 |
| chr18:61570503 | SERPINB2 | 0.219 |
| chr18:61570529 | SERPINB2 | 0.0007194 |
| chr18:61570551 | SERPINB2 | 0.004317 |
| chr18:61570609 | SERPINB2 | 0.0007194 |
| chr18:61570632 | SERPINB2 | 0.007914 |
| chr18:61575047 | SERPINB10 | 0.002158 |
| chr18:61575090 | SERPINB10 | 0.182 |
| chr18:61575120 | SERPINB10 | 0.002882 |
| chr18:61575126 | SERPINB10 | 0.028 |
| chr18:61575213 | SERPINB10 | 0.223 |
| chr18:61575232 | SERPINB10 | 0.0007215 |
| chr18:61575434 | SERPINB2 | 0.23 |
| chr18:61575572 | SERPINB2 | 0.033 |
| chr18:61582452 | SERPINB10 | 0.002907 |
| chr18:61582493 | SERPINB10 | 0.174 |
| chr18:61582497 | SERPINB10 | 0.183 |
| chr18:61582751 | SERPINB10 | 0.0007184 |
| chr18:61582816 | SERPINB10 | 0.212 |
| chr18:61582867 | SERPINB10 | 0.0007236 |
| chr18:61583071 | SERPINB10 | 0.19 |
| chr18:61583073 | SERPINB10 | 0.0007246 |
| chr18:61583083 | SERPINB10 | 0.003026 |
| chr18:61584529 | SERPINB10 | 0.205 |
| chr18:61584642 | SERPINB10 | 0.013 |
| chr18:61584726 | SERPINB10 | 0.029 |
| chr18:61584817 | SERPINB10 | 0.002551 |
| chr18:61584875 | SERPINB10 | 0.224 |
| chr18:61584982 | SERPINB10 | 0.019 |
| chr18:61585047 | SERPINB10 | 0.0007194 |
| chr19:859689 CFD | 0.006475 | |
| chr19:859793 CFD | 0.33 | |
| chr19:859831 CFD | 0.044 | |
| chr19:860325 CFD | 0.0007236 | |
| chr19:860751 CFD | 0.007194 | |
| chr19:860766 CFD | 0.002161 | |
| chr19:860787 CFD | 0.664 | |
| chr19:860852 CFD | 0.0007194 | |
| chr19:861056 CFD | 0.662 | |
| chr19:861102 CFD | 0.007246 | |
| chr19:861130 CFD | 0.007257 | |

| | | | |
|---|---|---|---|
| chr19:861140 | CFD | 0.019 | |
| chr19:861412 | CFD | 0.001443 | |
| chr19:861491 | CFD | 0.005029 | |
| chr19:861869 | CFD | 0.001437 | |
| chr19:861889 | CFD | 0.0007184 | |
| chr19:862009 | CFD | 0.406 | |
| chr19:862024 | CFD | 0.869 | |
| chr19:862801..862802 | | CFD | 0.657 |
| chr19:862912 | CFD | 0.016 | |
| chr19:863060 | CFD | 0.001439 | |
| chr19:863077 | CFD | 0.0007184 | |
| chr19:863179 | CFD | 0.0007184 | |
| chr19:863220 | CFD | 0.001437 | |
| chr19:863244 | CFD | 0.097 | |
| chr19:863356 | CFD | 0.325 | |
| chr19:863388 | CFD | 0.0007194 | |
| chr19:863402 | CFD | 0.328 | |
| chr19:863435..863436 | | CFD | 0.017 |
| chr19:863500 | CFD | 0.063 | |
| chr19:6677799 | | C3 | 0.483 |
| chr19:6677989 | | C3 | 0.0007184 |
| chr19:6678032 | | C3 | 0.0007184 |
| chr19:6678234 | | C3 | 0.053 |
| chr19:6678474 | | C3 | 0.0007184 |
| chr19:6678661 | | C3 | 0.003592 |
| chr19:6678701 | | C3 | 0.479 |
| chr19:6678753 | | C3 | 0.224 |
| chr19:6678871 | | C3 | 0.0007184 |
| chr19:6679254 | | C3 | 0.743 |
| chr19:6679360 | | C3 | 0.5 |
| chr19:6679563 | | C3 | 0.059 |
| chr19:6679645 | | C3 | 0.061 |
| chr19:6679773 | | C3 | 0.001437 |
| chr19:6680143 | | C3 | 0.0007184 |
| chr19:6680223 | | C3 | 0.0007194 |
| chr19:6680413 | | C3 | 0.063 |
| chr19:6680510 | | C3 | 0.481 |
| chr19:6681671 | | C3 | 0.004317 |
| chr19:6681925 | | C3 | 0.122 |
| chr19:6681991 | | C3 | 0.002158 |
| chr19:6682114 | | C3 | 0.0007194 |
| chr19:6682144 | | C3 | 0.164 |
| chr19:6684142 | | C3 | 0.136 |
| chr19:6684197 | | C3 | 0.0007194 |
| chr19:6684280 | | C3 | 0.005036 |
| chr19:6684297 | | C3 | 0.0007174 |
| chr19:6684380 | | C3 | 0.0007184 |
| chr19:6684901 | | C3 | 0.0007184 |
| chr19:6685211 | | C3 | 0.463 |
| chr19:6685304 | | C3 | 0.055 |
| chr19:6685930 | | C3 | 0.479 |

| | | |
|---|---|---|
| chr19:6685945 | C3 | 0.0007215 |
| chr19:6685953 | C3 | 0.429 |
| chr19:6685983 | C3 | 0.051 |
| chr19:6686089 | C3 | 0.022 |
| chr19:6686421 | C3 | 0.019 |
| chr19:6686504 | C3 | 0.022 |
| chr19:6686578 | C3 | 0.862 |
| chr19:6686659 | C3 | 0.858 |
| chr19:6687148 | C3 | 0.0007375 |
| chr19:6690352 | C3 | 0.172 |
| chr19:6690460 | C3 | 0.371 |
| chr19:6690461 | C3 | 0.171 |
| chr19:6690805 | C3 | 0.17 |
| chr19:6690966 | C3 | 0.168 |
| chr19:6690982 | C3 | 0.171 |
| chr19:6692790 | C3 | 0.171 |
| chr19:6692796 | C3 | 0.096 |
| chr19:6693163 | C3 | 0.171 |
| chr19:6693240 | C3 | 0.171 |
| chr19:6693273 | C3 | 0.171 |
| chr19:6693317 | C3 | 0.172 |
| chr19:6693387 | C3 | 0.0007184 |
| chr19:6693437 | C3 | 0.177 |
| chr19:6693636 | C3 | 0.012 |
| chr19:6693654 | C3 | 0.182 |
| chr19:6693683 | C3 | 0.011 |
| chr19:6694399 | C3 | 0.359 |
| chr19:6696178 | C3 | 0.014 |
| chr19:6696197 | C3 | 0.0007194 |
| chr19:6696267 | C3 | 0.582 |
| chr19:6697214..6697215 | C3 | 0.769 |
| chr19:6697406 | C3 | 0.0007184 |
| chr19:6697458 | C3 | 0.0007174 |
| chr19:6697590 | C3 | 0.02 |
| chr19:6697622 | C3 | 0.001439 |
| chr19:6697828 | C3 | 0.581 |
| chr19:6697829 | C3 | 0.001439 |
| chr19:6697870 | C3 | 0.0007194 |
| chr19:6697891 | C3 | 0.566 |
| chr19:6701838 | C3 | 0.575 |
| chr19:6701890 | C3 | 0.0007194 |
| chr19:6701947 | C3 | 0.0007194 |
| chr19:6701994 | C3 | 0.581 |
| chr19:6702022 | C3 | 0.581 |
| chr19:6702042 | C3 | 0.001439 |
| chr19:6702111 | C3 | 0.581 |
| chr19:6702157 | C3 | 0.0007194 |
| chr19:6702242 | C3 | 0.581 |
| chr19:6702246 | C3 | 0.171 |
| chr19:6702455 | C3 | 0.768 |
| chr19:6702598 | C3 | 0.008646 |

| | | |
|---|---|---|
| chr19:6702752 | C3 | 0.001439 |
| chr19:6706881 | C3 | 0.0007194 |
| chr19:6706967 | C3 | 0.0007194 |
| chr19:6707041 | C3 | 0.001437 |
| chr19:6707129 | C3 | 0.0007194 |
| chr19:6707237 | C3 | 0.0007194 |
| chr19:6707296..6707298 | C3 | 0.0007194 |
| chr19:6707323 | C3 | 0.0007194 |
| chr19:6707416 | C3 | 0.008633 |
| chr19:6707666 | C3 | 0.0007194 |
| chr19:6707900 | C3 | 0.002878 |
| chr19:6707913 | C3 | 0.0007194 |
| chr19:6708000 | C3 | 0.604 |
| chr19:6708107 | C3 | 0.588 |
| chr19:6708144..6708148 | C3 | 0.0007289 |
| chr19:6709419 | C3 | 0.06 |
| chr19:6709875 | C3 | 0.005036 |
| chr19:6709951 | C3 | 0.22 |
| chr19:6710145 | C3 | 0.279 |
| chr19:6710153 | C3 | 0.001439 |
| chr19:6710489 | C3 | 0.001439 |
| chr19:6710503 | C3 | 0.0007184 |
| chr19:6710729 | C3 | 0.135 |
| chr19:6710782 | C3 | 0.211 |
| chr19:6710948 | C3 | 0.001439 |
| chr19:6711245 | C3 | 0.143 |
| chr19:6711494 | C3 | 0.162 |
| chr19:6713262 | C3 | 0.237 |
| chr19:6713291 | C3 | 0.0007194 |
| chr19:6713399 | C3 | 0.0007194 |
| chr19:6713511 | C3 | 0.457 |
| chr19:6713713 | C3 | 0.051 |
| chr19:6713718 | C3 | 0.26 |
| chr19:6713736 | C3 | 0.039 |
| chr19:6713740 | C3 | 0.223 |
| chr19:6713752..6713757 | C3 | 0.423 |
| chr19:6713763..6713764 | C3 | 0.577 |
| chr19:6713764..6713765 | C3 | 0.701 |
| chr19:6713791..6713792 | C3 | 0.11 |
| chr19:6713793 | C3 | 0.402 |
| chr19:6713798 | C3 | 0.395 |
| chr19:6713799..6713810 | C3 | 0.157 |
| chr19:6713805 | C3 | 0.083 |
| chr19:6713810..6713811 | C3 | 0.095 |
| chr19:6713822..6713823 | C3 | 0.375 |
| chr19:6713826..6713829 | C3 | 0.263 |
| chr19:6713827..6713829 | C3 | 0.461 |
| chr19:6713829 | C3 | 0.368 |
| chr19:6713833..6713834 | C3 | 0.5 |
| chr19:6713839..6713841 | C3 | 0.516 |
| chr19:6713856..6713857 | C3 | 0.014 |

| | | |
|---|---|---|
| chr19:6714035 | C3 | 0.0007194 |
| chr19:6714109 | C3 | 0.0007174 |
| chr19:6714272 | C3 | 0.001435 |
| chr19:6714374 | C3 | 0.0007194 |
| chr19:6717956 | C3 | 0.299 |
| chr19:6718078 | C3 | 0.001437 |
| chr19:6718146 | C3 | 0.169 |
| chr19:6718387 | C3 | 0.0007174 |
| chr19:6718543 | C3 | 0.003125 |
| chr19:6718946 | C3 | 0.618 |
| chr19:6718992..6718993 | C3 | 0.0007194 |
| chr19:6719090 | C3 | 0.019 |
| chr19:6719453 | C3 | 0.007902 |
| chr19:6719527 | C3 | 0.199 |
| chr19:6719669..6719670 | C3 | 0.0007184 |
| chr19:6720575 | C3 | 0.001437 |
| chr19:6720601 | C3 | 0.002874 |
| chr19:6720644 | C3 | 0.003592 |
| chr19:6720720 | C3 | 0.00431 |
| chr19:6720723 | C3 | 0.68 |
| chr19:47812900 | C5AR1 | 0.0007184 |
| chr19:47813047 | C5AR1 | 0.314 |
| chr19:47813165 | C5AR1 | 0.003597 |
| chr19:47813335 | C5AR1 | 0.001437 |
| chr19:47822997 | C5AR1 | 0.994 |
| chr19:47823038 | C5AR1 | 0.0007174 |
| chr19:47823085 | C5AR1 | 0.0007174 |
| chr19:47823349 | C5AR1 | 0.994 |
| chr19:47823484 | C5AR1 | 0.994 |
| chr19:47823871 | C5AR1 | 0.005755 |
| chr19:47824281 | C5AR1 | 0.894 |
| chr19:47824323..47824324 | C5AR1 | 0.004304 |
| chr19:47844022 | C5AR2 | 0.0007174 |
| chr19:47844502 | C5AR2 | 0.005036 |
| chr19:47844653 | C5AR2 | 0.007205 |
| chr19:47845286..47845289 | C5AR2 | 0.013 |
| chr20:23064370 | CD93 | 0.0007194 |
| chr20:23064377 | CD93 | 0.003592 |
| chr20:23064558 | CD93 | 0.011 |
| chr20:23064912 | CD93 | 0.406 |
| chr20:23065209 | CD93 | 0.681 |
| chr20:23065342 | CD93 | 0.0007194 |
| chr20:23065528 | CD93 | 0.002874 |
| chr20:23065584 | CD93 | 0.0007194 |
| chr20:23065689 | CD93 | 0.0007184 |
| chr20:23065708 | CD93 | 0.021 |
| chr20:23065733 | CD93 | 0.0007184 |
| chr20:23065757 | CD93 | 0.067 |
| chr20:23065879 | CD93 | 0.005029 |
| chr20:23066095 | CD93 | 0.087 |
| chr20:23066338 | CD93 | 0.0007194 |

| | | |
|---|---|---|
| chr20:23066665 | CD93 | 0.0007194 |
| chrX:47483965 | CFP | 0.0007194 |
| chrX:47483966 | CFP | 0.784 |
| chrX:47485245 | CFP | 0.0007194 |
| chrX:47485378 | CFP | 0.0007184 |
| chrX:47485643 | CFP | 0.0007184 |
| chrX:47486050 | CFP | 0.005029 |
| chrX:47486217 | CFP | 0.218 |
| chrX:47486486 | CFP | 0.0007194 |
| chrX:47486558 | CFP | 0.001437 |
| chrX:47487077 | CFP | 0.0007194 |
| chrX:47487209 | CFP | 0.005036 |
| chrX:47487709 | CFP | 0.006466 |
| chrX:47489443 | CFP | 0.014 |
| chrX:47489471 | CFP | 0.423 |
